# Engineering Liquid Hierarchical Materials with DNA‐Programmed Spherical Nucleic Acids

**DOI:** 10.1002/advs.202504471

**Published:** 2025-06-05

**Authors:** Zeyu Chen, Xu Chen, Dan Lu, Huating Kong, Jingyi Ye, Chunhai Fan, Honglu Zhang, Huan Zhang

**Affiliations:** ^1^ School of Agriculture and Biology Shanghai Jiao Tong University Shanghai 200240 China; ^2^ School of Sencing Science and Enginnering, School of Electronic Information and Electrical Engineering Shanghai Jiao Tong University Shanghai 200240 China; ^3^ Shanghai Synchrotron Radiation Facility Shanghai Advanced Research Institute Chinese Academy of Sciences Shanghai 201204 China; ^4^ School of Chemistry and Chemical Engineering New Cornerstone Science Laboratory Frontiers Science Center for Transformative Molecules National Center for Translational Medicine Shanghai Jiao Tong University Shanghai 200240 China

**Keywords:** condensate droplets, hierarchical materials, phase separation, spherical nucleic acids

## Abstract

Inspired by nature, the orchestration of self‐assembling building blocks into hierarchical superstructures offers a transformative approach to functional materials design. While significant advances have been made in engineering solid‐state hierarchical materials such as crystals and superlattices, creating dynamic, liquid‐like hierarchical materials remains a profound challenge. Herein, a universal and efficient method is introduced to construct spherical nucleic acids (SNAs) functionalized with diverse nucleic acids (NAs), including random DNA sequences, circular DNA (circ‐DNA), single guide RNA (sgRNA), messenger RNA (mRNA), and multi‐branched DNA independent of sequence, length, or topology. By examining spatial configuration and mechanical rigidity in DNA‐mediated bonding, precise hierarchical assembly of SNAs is enabled. Furthermore, using these multivalent SNAs as programmable molecule equivalents, liquid‐phase hierarchical materials via phase separation are successfully created, forming microscale SNA droplets. These metal condensates exhibit dynamic liquid‐like properties and stimuli‐responsiveness, including enhanced photothermal effects in living cells. Our findings provide fundamental insights into the formation and dynamics of liquid hierarchical materials, offering potentials for designing living‐matter‐inspired systems and advancing applications in biomedicine and responsive materials.

## Graphical abstract

Programmable spherical nucleic acids assembly strategies for constructing hierarchical structures through engineered DNA bond to form novel liquid‐like metal droplets.

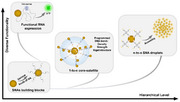

## 1 Introduction

The self‐assembly of intricate hierarchical structures, from atoms to molecules and beyond, is a foundational process in nature.^[^ ^1^, ^2^, ^3^ ^]^ Significant advancements have been made in engineering solid‐state hierarchical materials, such as crystals and superlattices, which rely on strong, directional interactions between densely packed building blocks.^[^ ^1^, ^4^, ^5^, ^6^, ^7^ ^]^ In contrast, recent developments in biomolecular condensates, driven by liquid‐liquid phase separation (LLPS),^[^ ^8^, ^9^, ^10^, ^11^, ^12^, ^13^ ^]^ have inspired new approaches for engineering liquid‐phase materials. The synthetic condensates leverage multivalent interactions involving polymers, supermolecules, proteins, or nucleic acids (NAs) to create dynamic liquid‐phase materials with living‐matter‐like properties, such as fluidity, adaptability, and responsiveness.^[^ ^14^, ^15^, ^16^, ^17^ ^]^ However, the exploration of liquid‐phase hierarchical materials remains largely unexplored.

Advances in DNA nanotechnology have transformed DNA‐functionalized micro‐ or nanoparticles into versatile building blocks for hierarchical assembly. Among these, spherical nucleic acids (SNAs), gold nanoparticles (AuNPs) functionalized with DNA, have emerged as a representative programmable atom equivalents (PAEs).^[^ ^6^, ^18^, ^19^ ^]^ The programmability of DNA enables precise geometries and addressability at the nanometer scale, allowing for sequence‐specific and tunable interactions.^[^ ^20^, ^21^ ^]^ This control over bonding interactions offers SNAs with exceptional tunability in both structure and function.^[^ ^22^, ^23^, ^24^, ^25^ ^]^ Despite this potential, achieving the precise manipulation of DNA‐mediated bonds to organize SNAs into liquid‐phase hierarchical materials remains a significant challenge.

Traditional and recently developed strategies for SNA assembly often require thiol modifications or specific nucleotide sequences, such as polyA/T/U, which increase the complexity and restrict their applicability to certain nucleic acids.^[^ ^26^, ^27^, ^28^, ^29^ ^]^ A universal and efficient assembly approach that accommodates nucleic acids regardless of sequence, topology, or type would greatly expand the utility of SNAs as modular building blocks. Additionally, a lack of systematic studies on factors influencing DNA‐mediated bonding, such as spatial configuration and mechanical rigidity, hinders the precise hierarchical assembly of SNAs.

The development of liquid‐phase hierarchical materials is also inspired by the unique properties of biomolecular condensates. These dynamic systems, formed via LLPS, exhibit inherent fluidity and adaptability, making them promising for applications in cytomimetics, sensing, drug delivery, and chemical microreactors.^[^ ^12^, ^30^, ^31^, ^32^ ^]^ DNA and RNA molecules, with their programmability and molecular recognition capabilities, have been used to construct all‐DNA or all‐RNA condensates through multivalent interactions. Such condensates serve as valuable analogs for studying phase separation phenomena. Recent studies have demonstrated the assembly of the complexes of AuNPs^[^ ^9^, ^33^ ^]^ or gold nanoclusters ^[^ ^34^ ^]^ assisted by metal ions or non‐specific interactions, displaying gel‐like behaviors. However, the integration of DNA‐programmed building blocks, such as SNAs, into liquid hierarchical superstructures remains unexplored. This gap represents an exciting opportunity to merge the programmability of DNA with the dynamic behavior of liquid‐phase materials.

Here, we address these challenges by introducing a universal and efficient method to construct SNAs functionalized with diverse nucleic acids, including random DNA sequences, circular DNA (circ‐DNA), single guide RNA (sgRNA), messenger RNA (mRNA) and multi‐branched DNA structures, regardless of their sequence, length, topology, or conformation. We systematically investigate the factors influencing DNA‐mediated bonding on AuNPs, focusing on spatial configuration and mechanical rigidity, to enable precise hierarchical assembly of SNAs. Beyond the assembly process, we demonstrate the formation of liquid‐phase hierarchical superstructures, specifically microscale SNA condensate droplets, driven by multivalent interactions. These metal condensates exhibit liquid‐like properties, dynamic behavior, and enhanced photothermal responsiveness in living cells, offering insights into their formation mechanisms and biological potential.

## 2 Results and Discussion

### 2.1 Assembly of Functionalized SNAs with Diverse Nucleic Acids

**Figure 1:**
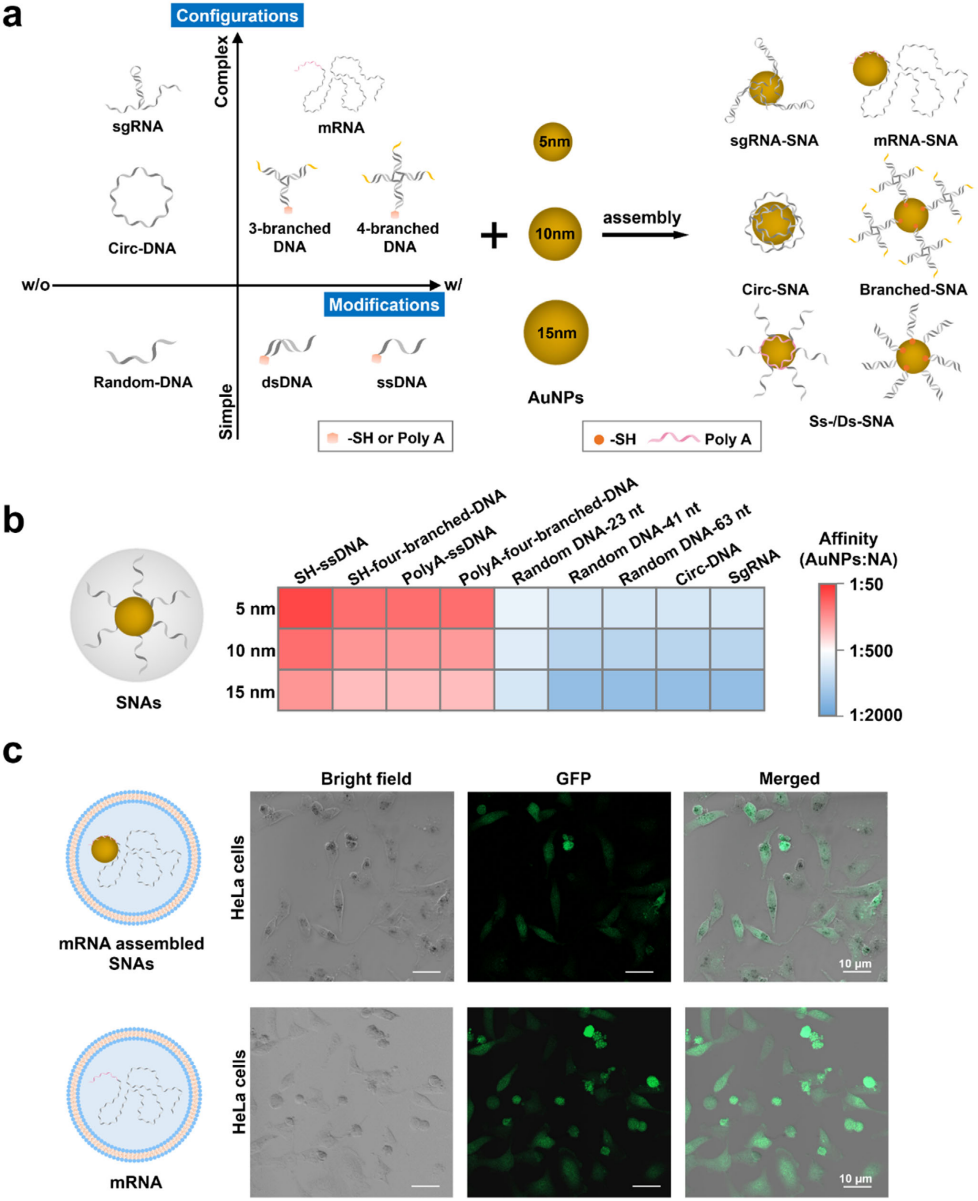
a) Schematic illustration of attaching various nucleic acids to AuNPs for the construction of SNAs. b) Heatmap depicting the affinity of different types of nucleic acids for AuNPs. c) Expression of GFP in HeLa cells after transfection with mRNA and mRNA‐assembled SNAs, indicating that the mRNA retains its biological function. Scale bars: 10 µm.

Developing an efficient SNAs assembly method with complex and functional nucleic acid structures, including circular nucleic acids, long structured RNA molecules, and higher‐order DNA nanostructures is crucial, due to their critical roles in the construction of programmable superstructures, genetic regulation, and therapeutic applications.^[^ ^35^, ^36^ ^]^ The immiscibility of butanol plays a key role in effectively separating DNA molecules from water, as demonstrated by Deng et al., who reported a rapid and simple approach to assemble SNAs with high thiol‐modified DNA density.^[^ ^37^ ^]^ By leveraging the hygroscopic property of butanol, this method compresses the physical space to increase the local concentration of nucleic acid molecules, thereby promoting rapid and efficient covalent chemical reactions or physical adsorption between AuNPs and nucleic acids within a confined reaction volume. Previous studies have demonstrated that the five nucleobases (A, T, U, C, and G) exhibit varying affinity toward AuNPs due to weak non‐covalent interactions with their amino and ketone functional groups, with A displaying the strongest affinity and T displaying the weakest.^[^ ^38^ ^]^ We hypothesized that the extremely crowded, high concentrations of nucleic acids and AuNPs during this process could facilitate weak interactions between unmodified nucleic acids and AuNPs, breaking the restrictions of nucleic acid type, sequence, modification, length, and topology. To investigate this hypothesis, we designed various nucleic acids and examined their assembly performance with AuNPs of different diameters (5, 10, and 15 nm, **Figure** **1**a). Our selections included: (1) thiolated single‐stranded (ss) and double‐stranded (ds) DNA as well as multi‐branched DNA capable of forming Au‐S bond with AuNPs; (2) polyA‐tailed ssDNA, dsDNA, multi‐branched DNA, and mRNA with a strong affinity for AuNPs; and (3) unmodified random sequence DNA, circular DNA, and sgRNA (see Figure S1, Supporting Information for electrophoresis characterization of nucleic acids used and Table S1, Supporting Information for detailed sequences), which typically have a low affinity for AuNPs. Of note, the lengths of these nucleic acids varied from 23 to ≈1 kb nucleotides.

We characterized the assembled SNAs using ultraviolet‐visible (UV–Vis) spectroscopy, dynamic light scattering (DLS), and agarose gel electrophoresis (AGE). The UV–Vis spectra revealed a red‐shift absorption peak compared to bare AuNPs, indicating successful nucleic acid assembly on AuNPs (Figure S2, Supporting Information). DLS results confirmed that the hydrodynamic size of all SNAs was larger than that of bare AuNPs (Table S2, Supporting Information). AGE further validated the successful assembly, with SNAs showing distinctive band positions (Figure S3, Supporting Information).

As shown in Figure 1b, the relative affinity of the representative nucleic acids (NAs) for AuNPs was defined as the minimum AuNPs: NA ratio required to form stable SNAs (Table S3, Supporting Information). As established in our previous study,^[^ ^39^ ^]^ we assume that a lower stoichiometric ratio required to form stable SNAs implies that the nucleic acids have stronger interaction with AuNPs, thus suggesting a higher affinity toward AuNPs. It is noteworthy that certain nucleic acid sequences, such as mRNA and sgRNA, feature double‐stranded or partially secondary structures. As expected, thiol‐modified nucleic acids exhibited the highest affinity, followed by polyA‐tailed nucleic acids. Random DNA without specific modifications showed relatively lower affinity, while circ‐DNA and sgRNA, which possess secondary conformations, exhibited the lowest affinity. Across various DNA sequences, the affinity for AuNPs followed the order: SH‐DNA > polyA‐DNA > random‐sequence DNA> DNA or RNA with secondary conformations. For different nucleic acid structures, linear single‐stranded nucleic acids had the highest affinity, followed by linear double‐stranded and circular single‐stranded nucleic acids. Our experimental results revealed that while these structures did not prevent assembly, they did influence the efficiency of the process, likely due to the presence of fewer available interacting groups, which hindered their binding to AuNPs. Moreover, as the length of the nucleic acids increased, assembly became more challenging. Meanwhile, the numbers of DNA per AuNPs were calculated, as illustrated in Figure S4 (Supporting Information), DNA with ‐SH modification showed the highest DNA/Au number (e. g. more than 150 for 15 nm AuNPs formed SNAs). More importantly, the polyA length can significantly regulate the DNA/Au number, from tens or hundreds with polyA10, to 2‐4 DNA per AuNPs (5 nm) with polyA80 (Figure S4, Supporting Information).

An exciting revelation was the ability to attach RNA, such as mRNA and sgRNA, with specific biological functions to AuNPs without requiring any modifications. These RNA‐based SNAs were found to be stable, and we investigated whether the RNA reserves its functionality. mRNA is observed in close proximity to the AuNPs, as indicated by the stain (Figure S5, Supporting Information). As shown in Figure 1c and Figure S6 (Supporting Information), mRNA‐assembled SNAs demonstrated high stability in the presence of the RNA transfection reagent (Xfect™ RNA Transfection Reagent) and were successfully delivered into HeLa cells. The mRNA‐assembled SNAs expressed comparable levels of the encoded green fluorescent protein (GFP) to those observed in the mRNA‐only group (Figure S7, Supporting Information), indicating that the mRNA retained its functionality. However, without the transfection reagent, GFP expression was not observed, likely due to SNAs being trapped in endosomes rather than reaching the cytoplasm (Figure S8, Supporting Information).

### 2.2 Spatial Configuration and Mechanical Rigidity of DNA Bonds Programmed Hierarchical Assembly of SNAs

SNAs with active sticky ends represent promising building blocks for the rational construction of intricate hierarchical structures.^[^ ^6^ ^]^ However, efficient construction of such hierarchical structures requires precise control over surface DNA bonds, a critical challenge due to the steric hindrance and electrostatic repulsion arising from the densely grafted DNA shell on the nanoparticle surface. This dense DNA layer creates a crowded and charged environment that influences the accessibility and reactivity of binding sites. To address this, we investigated how the spatial configuration and mechanical rigidity of DNA binding sites affect the efficiency of hierarchical assembly using SNA as programmable “atom equivalents”.

**Figure 2:**
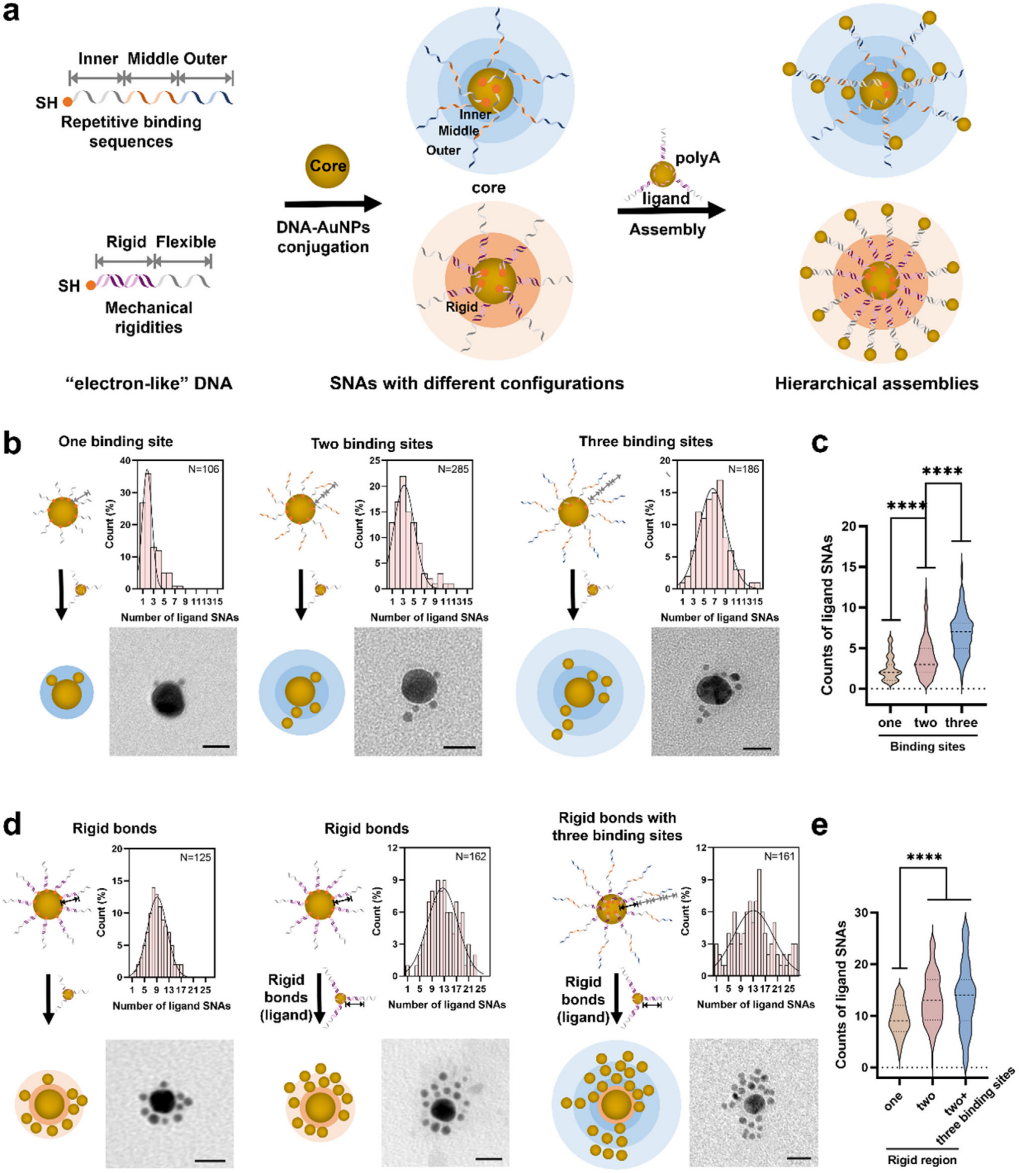
a) Schematic representation of the spatial configuration and the rigidity of DNA bonds designed on SNA core (15 nm) and ligands (5 nm) for hierarchical assembly of SNAs. b) Illustration and representative TEM images of hierarchical structures assembled using repetitive binding sites, and c) statistical analysis of the number of bound ligands in different groups. d) Illustration and representative TEM images of hierarchical structures assembled using rigid DNA bonds, and e) statistical analysis of the number of bound ligands in different groups. The black curve represents a Gaussian distribution fit. Scale bars: 20 nm. The significance is indicated as follows: ^****^, *P* < 0.0001, one‐way analysis of variance (ANOVA).

In our experiments, to construct the hierarchical structure, 15 and 5 nm AuNPs were conjugated with DNA molecules to form core and ligand SNAs that would interact with each other through the hybridization between binding sites (15 nt DNA sequence) on the bond of each component. To examine the effect of the spatial configuration of binding sites on the assembly efficiency, we designed a series of DNA bonds with repetitive binding sites and a rigid duplex that extends vertically from the AuNPs surface, positioning the DNA binding sites from proximal to distal locations. Additionally, polyA sequences (polyA‐DNA) of varying lengths were used as DNA bonds to regulate the lateral spacing and density of binding sites on the SNA surface, thereby reducing the steric hindrance for further hybridization, as previously reported.^[^ ^40^, ^41^ ^]^ As shown in **Figure** **2**a, DNA designed with one‐, two‐, and three‐repetitive binding sites (with 15, 30 and 45 nt length, respectively) was assembled on a 15 nm SNAs core (upper panel). Meanwhile, a mechanically rigid dsDNA region (15 bp) near AuNPs surface was introduced to facilitate the extension of binding sites on SNAs core (lower panel).

Transmission electron microscopy (TEM) images and the statistical analysis of the number of ligands on each core were employed to explore the effect of the spatial configuration of DNA bonds on hierarchical assembly and evaluate the efficiency. Based on our results, for better comparison, the SNA cores shown in Figure 2 are assembled with SH‐modified DNA, while the SNA ligands are assembled with polyA50‐tailed DNA. As illustrated in Figure 2b, the number of assembled SNA ligands on each core increased significantly with the number of binding sites on the core. Specifically, the median number of polyA50 constructed ligands increased from 1 (with one binding site) to 3 (with two binding sites) and reached 7 with three binding sites (Figure 2b,c and Table S4, Supporting Information). TEM images further confirmed that the SNA ligands were arranged in one, two, and three layers around the core (Figure 2b; Figures S9 and S10a, Supporting Information). We also conducted a quantitative analysis of the number of SNAs ligand bound to each layer of SNAs core with multiple binding sites. As results shown in Figure S11 (Supporting Information), with an increase of repeated binding sites (from one to three), the number of assembled SNA ligands on each core increased and exhibited one, two, and three layers around the core in accordance. Moreover, SNA cores with three repetitive binding sites exhibited higher assembly efficiency compared to those assembled with random DNA sequences (Figures 2b and S10b, Supporting Information). These results suggest that introducing multiple binding sites on the SNA core provides additional regions for ligand recognition, thus enhancing the efficiency of hierarchical assembly.

Next, we adjusted the mechanical rigidity of the DNA bonds by introducing a dsDNA region (15 bp) near the AuNP surface. This adjustment facilitated the extension of binding sites (15 nt) on SNAs for hierarchical assembly (Figure 2a,d). The rigid DNA region was formed by incubating SH‐DNA with a complementary sequence, leaving the remaining single‐stranded binding site available for further assembly (see detailed DNA sequences in Table S1, Supporting Information). TEM images and statistical data (Figure 2d and Figure S12, Supporting Information) revealed that introducing the rigid dsDNA region near the core surface significantly increased the number of SNA ligands (Figure 2d,e). Notably, for polyA50 constructed SNA ligands, the median number of ligands bound to each core reached 9, surpassing the assembly of 6 with three repetitive binding sites (Figure 2d; Figure S12 and Table S4, Supporting Information). This improved efficiency demonstrates the critical impact of DNA bond rigidity on the hierarchical assembly of SNAs. Motivated by these results, we further introduced the rigid dsDNA region on the SNA ligands. This modification further enhanced assembly efficiency, with the median number of bound ligands bound reaching 12 when both the SNA core and ligands were constructed with rigid DNA bonds (Figure 2d, second column). We attribute these results to the spatial configuration of DNA bonds on SNAs. The rigid dsDNA region prevents DNA molecules from adhering to the AuNP surface, allowing the binding sites to extend outward even at high DNA densities. This extended configuration enhances the interaction between the DNA binding sites on the SNA core and ligands, thereby improving the efficiency of hierarchical assembly.

Optimizing the configuration of multiple active binding sites and incorporating rigid dsDNA bonds resulted in the highest assembly efficiency, with a medium number of 13 ligands per core (Figure 2d,e, third column, Figure S12 and Table S4, Supporting Information). Overall, programmable regulation of the spatial configuration and mechanical rigidity of DNA bonds‐ specifically increasing the number of binding sites and replacing flexible ssDNA bonds with rigid dsDNA enabled the efficient assembly of hierarchical structures (Figures S13–S15 and Table S4, Supporting Information).

### 2.3 DNA‐Programmed Assembly of SNAs into Microscale Liquid Hierarchical Superstructures

**Figure 3:**
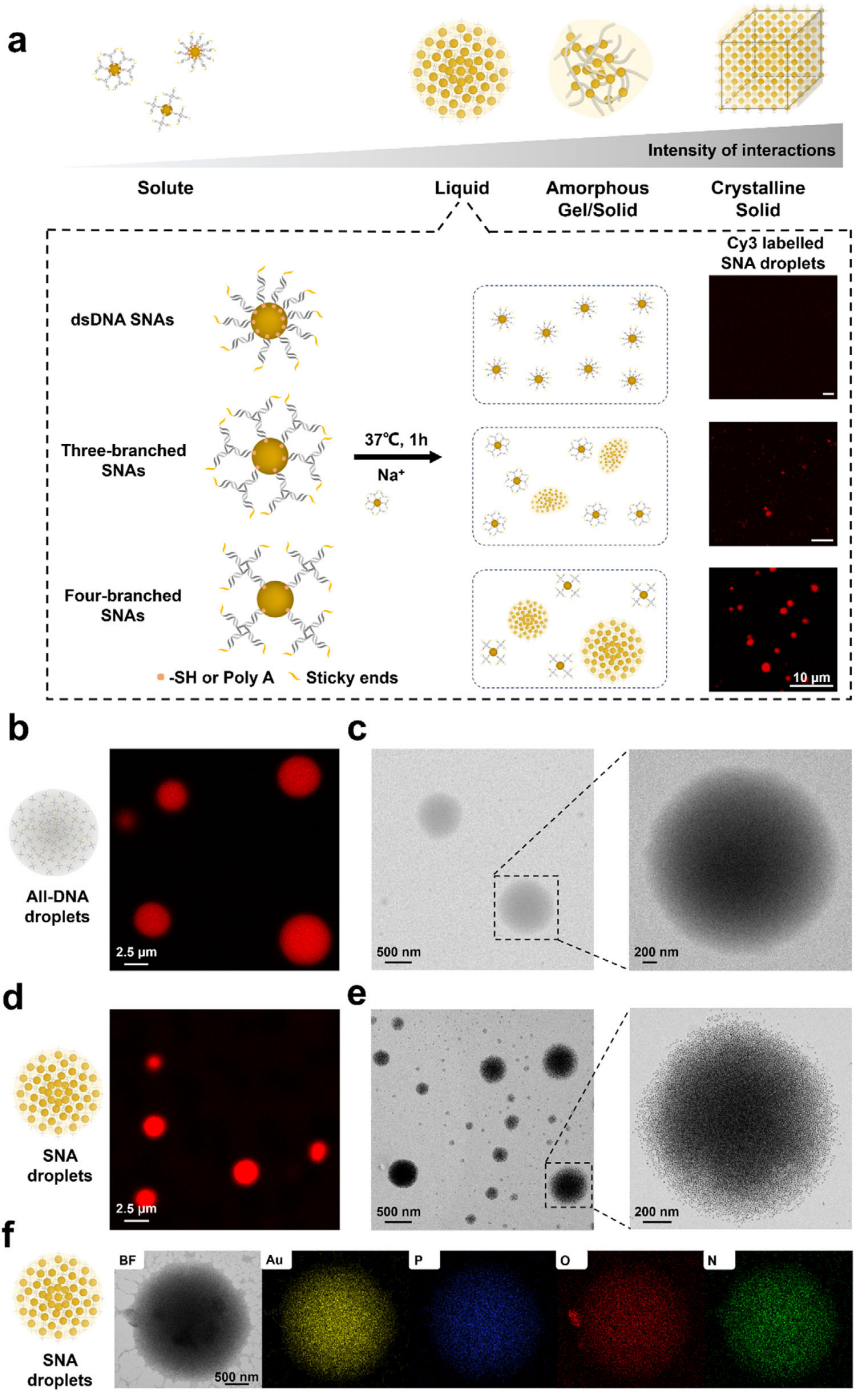
a) Schematic diagram and representative CLSM images illustrating the influence of DNA with different multivalency conformations, two‐branched (dsDNA), three‐branched, and four‐branched DNA nanostructures on the construction of SNA droplets. Scale bars: 10 µm. Representative CLSM and TEM images of b,c) all‐DNA droplets and d,e) SNA droplets (10 nm). f) HRTEM image of SNA droplets (5 nm) and the corresponding EDS mapping profiles for Au, P, O, and N elements.

Motivated by recent advancements in subcellular biomolecular condensates, we explore to transition SNAs‐based superstructures from solid to liquid phase, employing multivalent and weakened DNA bonds on SNAs (**Figure** **3**a). To evaluate the interaction efficacy of SNAs, we started this work by designing various SNAs with different multivalency conformations, two‐branched (dsDNA), three‐branched, and four‐branched DNA nanostructures (Figure 3a). One branch of these nucleic acids was modified with thiol or polyA sequence to construct SNAs, leaving other branches with sticky ends of palindromic sequences for self‐association during hierarchical assembly (see detailed sequences in Table S1, Supporting Information). Therefore, the bond valency of each branched DNA on SNAs was one, two, and three, respectively. Each DNA structure was formed and characterized (Figure S1, Supporting Information) before attaching onto AuNPs. The formed multi‐branched DNA was then assembled onto AuNPs (5 and 10 nm) to construct higher‐order, muti‐branched SNAs using the aforementioned method.^[^ ^42^ ^]^ and characterized by UV–Vis spectrometer (Figure S16, Supporting Information). Microscale liquid hierarchical superstructures were constructed using muti‐branched SNAs in NaCl solution (see details in the methods section).

A confocal laser scanning microscope (CLSM) was used to characterize the products of muti‐branched SNAs assembly. We unified the concentration of the branched DNA on AuNPs to 1 µm, ensuring that all systems had the same DNA content. As shown in Figure 3a, the formation of spherical‐shaped condensate droplets (2 to 3 µm) was observed when using four‐branched DNA. In comparison, samples of three‐branched SNAs only yielded small, irregularly shaped aggregates (<1 µm), while no aggregates were found in samples of dsDNA‐constructed (two‐branched) SNAs. These differences are attributed to the stronger interactions among the four‐branched SNAs, arising from the increased valency (number of sticky ends) associated with four‐branched DNA bonds. Conversely, insufficient interactions were observed in three‐ and two‐branched SNAs due to their lower number of sticky ends.

Further morphological analysis of condensate droplets was performed using TEM and CLSM. We compared SNA droplets formed by four‐branched DNA assembled on 5 and 10 nm AuNPs, along with all‐DNA droplets formed by four‐branched DNA. TEM and CLSM images revealed that SNA droplets, whether formed with 5 nm or 10 nm AuNPs, exhibited symmetrically spherical morphologies similar to all‐DNA droplets, with homogeneously dispersed DNA and AuNPs (Figure 3b–e, Figures S17 and S18, Supporting Information). Furthermore, SNAs constructed from four‐branched DNA tagged with thiol (SH), polyA sequences, or random sequences (rs) all formed condensate droplets (Figure S17, Supporting Information). Previous studies have shown that the hybridization strength between DNA strands is sensitive to salt concentration, which influences the binding probability of DNA sticky ends.^[^ ^42^, ^43^, ^44^, ^45^ ^]^ In our study, we investigated how SNAs (constructed by thiol tagged four‐branched DNA) concentration and salt concentration impact the formation of SNA droplets. Consistent with findings of all‐DNA droplets,^[^ ^43^ ^]^ results in the phase diagram (Figure S19, Supporting Information) demonstrated that the formation of SNA droplets is significantly influenced by these parameters. These findings suggest that the density and spatial configuration of valency contributed by the multi‐branched DNA bonds, as well as SNAs and salt concentrations, influence the formation and size of SNA condensate droplets.

It is noteworthy that the TEM images of SNA droplets (Figure 3e and Figure S18, Supporting Information) showed dense structures with high contrast, indicating the presence of numerous AuNPs within the droplets, compared to the all‐DNA droplets. High resolution TEM (HRTEM) and energy‐dispersive spectroscopy (EDS) elemental mapping images (Figure 3f) further revealed a higher relative abundance of gold (Au), nitrogen (N), oxygen (O), and phosphorus (P) elements in the SNA droplets. In addition, we characterized the SNA droplets using a scanning transmission electron microscope (STEM). As shown in Figure S20 (Supporting Information), the dried SNA droplets formed by 5 nm AuNPs demonstrated a spherical shape with a size of ≈2 µm. 3D reconstruction of these droplets using electron tomography techniques (Video S1, Supporting Information) confirmed the dense, 3D structures of the SNA droplets. Additionally, to further support our findings and provide more comprehensive structural insights, we conducted a z‐stack scanning of Cy3‐labelled all‐DNA and SNA droplets using CLSM. Figure S21 (Supporting Information) exhibited the 3D cross‐sectional images of both all‐DNA and SNA droplets, confirming that the droplets are 3D structures composed of densely packed DNA or SNAs. These findings confirm the successful formation of hierarchical structures, utilizing multi‐branched SNAs as programmable molecule equivalents.

### 2.4 Growth Mechanism and Liquid‐Like Behavior of SNA Droplets

**Figure 4:**
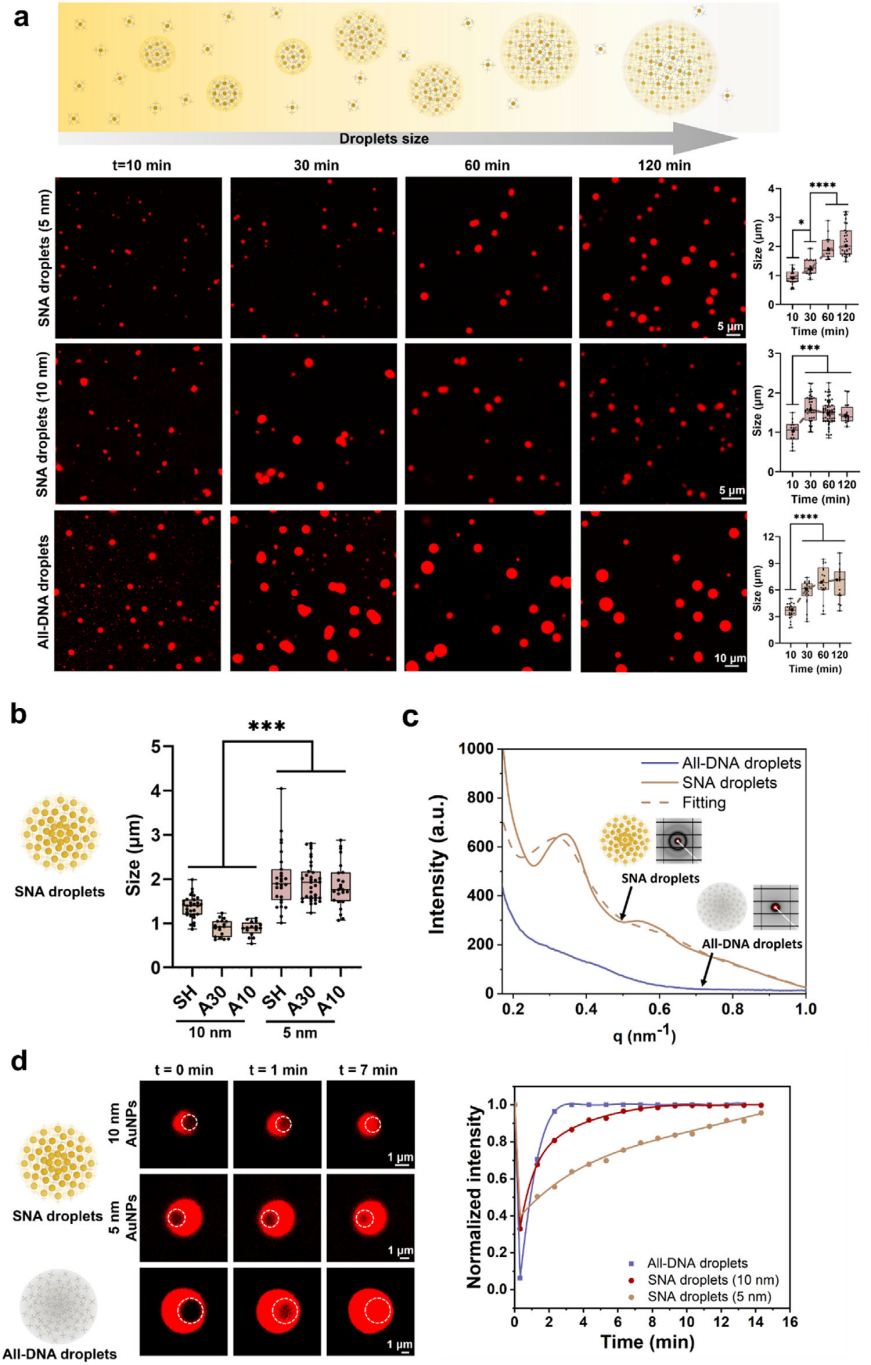
a) CLSM images demonstrated the growth of DNA and SNA droplets (5 nm, polyA30) at different time points, along with statistical analysis of the size distribution (the longest chord length of condensates, N=50). ^****^, *P* < 0.0001; ^***^, *P* < 0.001; ^**^, *P* < 0.01; ^*^, *P* < 0.1, one‐way ANOVA. Scale bars: 5 and 10 µm. b) The statistical analysis of the size distribution of droplets formed by various DNA molecules and AuNPs (5 nm and 10 nm). ^***^, *P*<0.001, one‐way ANOVA. c) The SAXS characterization of all‐DNA and SNA droplets, with an inserted dashed line showing the corresponding 2D scattering patterns. d) Representative CLSM images showing partially photobleached Cy3 labeled droplets at specific timepoints and the fluorescence recovery curve during the FRAP experiment in 15 min (left), and fluorescence recovery curves showing the different recovery rates of the partially photobleached all‐DNA and SNA droplets (right). Scale bars: 1 µm.

We investigated the growth process of the SNA droplets using CLSM. The characteristic size of droplets we measured includes both spherical and aggregated condensates. Specifically, we used the longest chord length of the condensates to represent changes in their size over time. As shown in **Figure** **4**a, the SNA droplets rapidly formed, reaching ≈1 µm in size within 10 min. Over the next hour, these droplets gradually grew to 2–3 µm, after which the growth rate slowed. In contrast, all‐DNA droplets displayed a similar growth behavior but at a faster rate, reaching 3 µm within 10 min and 6 µm after 1 h. Size distribution analysis showed that SNA droplets formed with 10 nm AuNPs were smaller than those formed with 5 nm AuNPs, and both were smaller than the all‐DNA droplets (Figure 4a,b and Figure S17, Supporting Information). This difference is potentially attributed to the monomer concentration and valency density within each droplet. Specifically, the concentration of four‐branched DNA used to construct all‐DNA and SNA droplets was 5, 3.3, and 1.1 µm, respectively (see Methods). In addition, the density of sticky ends is higher in all‐DNA droplets compared to SNA droplets due to the presence of AuNP cores and the rigid conjugation between DNA and AuNPs. Calculations (see methods) revealed a significant difference in valency density, defined as the number of sticky ends per unit volume of droplets, between SNA droplets. Specifically, 5 nm SNA droplets (0.17) exhibited a four‐fold higher valency density compared to 10 nm SNA droplets (0.042). This increased monomer concentration and valency density could potentially enhance the interaction possibilities, contributing to the formation of larger droplets. We further characterized the all‐DNA and SNA droplets using small angle X‐ray scattering (SAXS). The scattering peaks were observed for SNA droplets at scattering vectors (q) of 0.30, 0.53, and 0.78 nm^−1^ with the peak at 0.30 nm^−1^ being the sharpest, while all‐DNA droplets exhibited a weaker peak at q of 0.38 nm^−1^ (Figure 4c). Whereas, as shown in Figure S22 (Supporting Information), no scattering peaks were observed for SNAs. In addition, the inserted 2D scattering patterns revealed a highly‐ordered hierarchical structure composed of nanoparticles in the SNA droplets. This is consistent with our electron microscopy results (Figure 3e), which showed that SNAs inside the droplets are more tightly packed, while those in the outer layers exhibit a relatively loose arrangement, forming a 3D spherical structure.

To investigate the liquid‐like behavior of the SNA droplets, we conducted fluorescence recovery after photobleaching (FRAP) experiments (Figure 4d, Figure S23, Supporting Information), a standard technique used to study the mobility and diffusion of molecules within condensates. Using CLSM, we carried out the FRAP analysis of Cy3‐labeled SNA droplets. The results, shown in Figure 4d (see Videos S2 to S4, Supporting Information), revealed rapid and complete fluorescence recovery of Cy3 within the photobleached area (white circle), confirming the liquid‐like behavior of the assembled SNA droplets. Quantitative analysis of the FRAP results revealed that the half‐lives (t_1/2_) for all‐DNA droplets and SNA droplets formed with 10 and 5 nm AuNPs were ≈0.80, 1.62, and 7.15 min, respectively, with recovery efficiencies of ≈100.00%, 99.7%, and 92.9% (Figure 4d). The apparent diffusion coefficients (D_app_) were estimated at ≈2.9×10^−1^, 1.4×10^−2,^ and 7.3×10^−3^ µm^2^ min⁻¹ for all‐DNA droplets and SNA droplets formed with 10 nm and 5 nm AuNPs, indicating higher mobility for SNA droplets with 10 nm AuNPs than with 5 nm AuNPs. As mentioned earlier, compared to 10 nm SNA droplets, the sticky‐end density of 5 nm SNA droplets is four times higher (0.17 vs 0.042). This enhanced sticky‐end density likely strengthens the interactions of SNA monomers, thereby stabilizing the droplet architecture. This conclusion is further corroborated by SNA droplet sizes observed in Figure 4b, where the size of SNA droplets constructed by 5 nm AuNPs is larger than 10 nm. Concurrently, the intensified sticky‐end hybridization kinetics can be attributed to the suppressed droplet fluidity. In addition, we observed the fusion behavior of SNA (5 nm) and all‐DNA droplets (Figure S24, Supporting Information) and calculated the inverse capillary velocities, comparing them with previously studied.^[^ ^14^, ^43^, ^46^, ^47^, ^48^ ^]^ As summarized in Table S5 (Supporting Information), the inverse capillary velocities, indicative of fusion dynamics, were measured at 10.26 min µm^−1^ for SNA droplets and 2.07 min µm^−1^ for all‐DNA droplets, suggesting that all‐DNA droplets fuse more rapidly, reflecting their higher fluidity. This difference corresponds well to our findings from the droplet growth and FRAP experiments, which demonstrate higher molecular mobility in all‐DNA droplets compared to SNA droplets. Overall, SNA droplets exhibit similar physical properties to all‐DNA droplets, including spherical morphology, time‐dependent growth behavior, and characteristic liquid‐like fluidity. However, due to the presence of solid AuNPs, the growth rate, the final size, and the fluidity of SNA droplets are slower than those of all‐DNA droplets (Figure 4).

### 2.5 Enhanced Photothermal Responsiveness of SNAs Droplets in Living Cells

The inherent optical properties of AuNPs, particularly their surface plasmon resonance (SPR), enable in situ photothermal responses upon exposure to specific wavelengths of light.^[^ ^49^ ^]^ This characteristic is greatly promising for biomedical applications like photothermal therapy.^[^ ^50^, ^51^ ^]^ To leverage this property, we explored the photothermal responsiveness of our SNA droplets. CLSM imaging (Figure S25 and video S5, Supporting Information) showed that, upon exposure to 561 nm laser irradiation, partial blank areas appeared in the dark regions of the SNA droplets (5 nm). This observation suggests a laser‐induced photothermal effect on AuNPs (the plasmon absorption peak of AuNPs is ≈520 nm), where the AuNPs at the core of SNA droplets act as photothermal hotspots, locally melting DNA duplexes.^[^ ^52^ ^]^ and leading to the disassembly of DNA strands and detachment of AuNPs from the SNA droplets. Notably, when turning off the laser excitation, the blank areas disappeared, and the SNA droplets returned to their normal dark spherical form after 15 min. The high mobility of the free SNAs nanoparticles from the solute phase allowed them to diffuse readily and replenish defect sites within the droplets, as further confirmed by statistical analysis of the brightness of the SNA droplets (Figure S25, Supporting Information), indicating the detaching and recruiting of SNAs nanoparticles.

**Figure 5:**
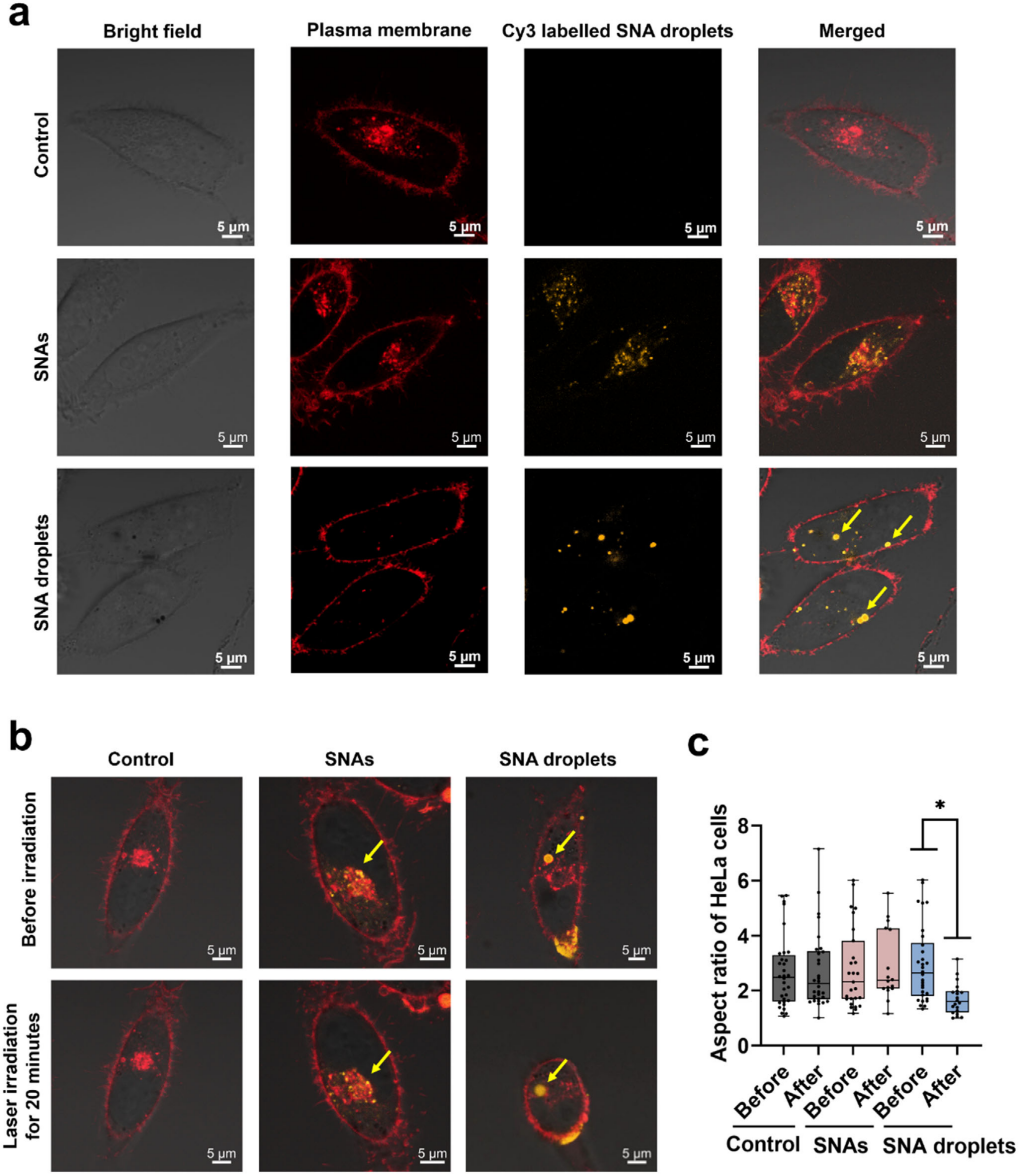
a) CLSM images showing the internalization of SNAs or SNA droplets into HeLa cells. The SNA droplets are indicated by the yellow arrows. Scale bars: 5 µm. b) Representative CLSM images of HeLa cells incubated without nanoparticles (left panel), with dispersed SNAs (middle panel), and with SNA droplets (right panel) before and after 20 min of exposure to 561 nm laser. The laser‐irradiated areas correspond to the SNA droplets. Scale bars: 10 µm. c) Statistical analysis of the aspect ratio of thirty HeLa cells before and after different treatments. Statistical significance is indicated as follows: ^*^, *P* < 0.1, one‐way ANOVA.

Building on the liquid‐like behavior and photothermal responsive properties, we introduced SNA droplets to HeLa cells to explore their photothermal effects on cellular behavior. Previous studies have shown that SNAs can be efficiently internalized by tumor cells through receptor‐mediated endocytosis.^[^ ^53^, ^54^ ^]^ and can escape from lysosomes to function within the cytoplasm.^[^ ^55^ ^]^ We first evaluated the cellular uptake of SNAs and SNA droplets. After incubating HeLa cells with SNAs or SNA droplets (with a final concentration of 50 nm) for 8 h, CLSM revealed clusters of orange fluorescence within the cells (**Figure** **5**a), indicating effective uptake of SNAs and SNA droplets. In contrast, the fluorescent dots in SNAs treated group were more dispersed, while there were large fluorescent dots ≈1–2 µm in SNAs droplets group, which indicated the internalization of SNA droplets. Statistical analysis of the average fluorescence intensity inside HeLa cells revealed comparable uptake efficiency of SNAs and SNA droplets (Figure S26, Supporting Information). We further investigated the uptake pathways of SNA droplets, and the results in Figure S27 (Supporting Information) indicated that the macropinocytosis inhibitor significantly reduced the uptake of SNA droplets, while other inhibitors (chlorpromazine for inhibition of clathrin, methyl‐β‐cyclodextrin for inhibition of caveolin) had little impact on the uptake efficiency. We attribute this to the size of SNA droplets. Typical uptake pathways involving clathrin or caveolin‐mediated endocytosis are usually associated with nanometer‐scale particles,^[^ ^55^, ^56^ ^]^ while the size of SNA droplets ranges from 1–3 µm.

Next, we examined the photothermal response of SNA droplets within the cells. As shown in Figure 5b, 20 min of 561 nm laser irradiation of cells containing SNA droplets led to the contraction of cellular protrusions and a more rounded cell shape, indicating significant morphological changes. In contrast, control groups and cells treated with dispersed SNAs showed no noticeable morphological changes under the same conditions (Figure 5b), suggesting that the laser had minimal adverse effects on these cells. Furthermore, we investigated the photothermal effect of all‐DNA droplets, and the results demonstrated that laser irradiation had no discernible impact on cell morphology (Figure S28, Supporting Information). Statistical analysis of the aspect ratio of Hela cells before and after laser irradiation showed greater changes in cells treated with dense SNA droplets compared to those with dispersed nanoparticles. To further validate the photothermal effect of SNA droplets, we assessed cell viability before and after laser irradiation for the blank control, SNAs, and SNA droplets using the cell counting kit‐8 (CCK‐8) assay (Figure S29, Supporting Information). The results showed that cell viability in the control group showed no change, and the cell viability of cells treated with dispersed SNAs slightly decreased, but no significant difference was observed. Notably, the viability of cells treated with SNA droplets was significantly decreased to 60% after 20 min of laser irradiation. This result is consistent with the observed morphological changes in cells and further emphasizes the enhanced photothermal performance of these highly dense SNA droplets compared to the dispersed SNAs. Previous studies have demonstrated that aggregated AuNPs exhibit enhanced photoacoustic imaging and photothermal therapy efficacy in cancer cells.^[^ ^57^, ^58^ ^]^ Therefore, we conjecture that the cell morphology change we observed is due to the heat generated by SNA droplets under light irradiation. We propose that the key difference lies in the density and localized heat generation within the SNA droplets. When SNAs are dispersed, they may not generate sufficient localized heating to cause significant morphological changes. However, when SNAs are packed together within a droplet, the higher concentration of AuNPs allows for enhanced heat generation, leading to more pronounced cellular effects upon photothermal activation. In all, this can be attributed to the enhanced photothermal effect from the condensed SNA droplets, highlighting their potential for effective photothermal therapy.

## 3 Conclusion

In this study, we developed a universal and efficient strategy for constructing SNAs functionalized with various nucleic acids, demonstrating adaptability to a range of characteristics, including nucleotide sequences, lengths, topologies, and conformations. Notably, the ability to directly conjugate biologically functional nucleic acids, including mRNA, sgRNA, and circular DNA, with nanoparticles, without additional modifications, is a significant advancement. These nucleic acids, each with distinct topologies and roles in fundamental biological processes such as protein synthesis, genetic information storage and transfer, and gene editing, can now be more effectively utilized in genetic regulation, diagnostics, and therapy.

Our assembly strategy also enables the construction of higher‐order hierarchical structures. By exploring DNA‐mediated bonding on SNAs, we identified the critical roles of spatial configuration and the mechanical rigidity of DNA bonds in enabling precise hierarchical assembly. Leveraging these insights, we successfully demonstrated the formation of microscale hierarchical superstructures in the liquid phase through phase separation, using multi‐branched SNAs as programmable molecule equivalents. These structures exhibited dynamic liquid‐like properties and enhanced photothermal responsiveness in living cells.

This DNA‐programmed approach to constructing nanoparticle condensate droplets provides a versatile platform for exploring functional liquid‐phase hierarchical materials. The feasibility of encoding stimuli‐responsive features into DNA‐programmed droplets enables precise control and activation in response to external physical and biochemical stimuli. Moreover, the inherent programmability of nucleic acids provides an effective means of regulating the assembly and phase separation processes, facilitating the customization of material functionalities. These liquid‐phase materials exhibit the potential to incorporate a wide range of functional components, including enzymes, drug molecules, or nanoparticles, thereby expanding their applicability in fields such as biomimetics, sensing, drug delivery, therapy, and chemical microreactors. Our findings bridge the gap between the programmability of DNA and the dynamic nature of liquid‐phase materials, providing a deeper understanding of their formation mechanisms and opening new avenues for designing biomimetic and active materials.

## Conflict of Interest

The authors declare no conflict of interest.

## Author Contributions

Z. C. and X. C. contributed equally to this work. H. Z. and H. Z. conceived the idea, designed the study, and supervised the project. Z. C. and X. C. conducted most of the experiments, data analysis, and wrote the manuscript. K. H. performed the SAXS experiments and analyzed the data. Z. C., H. Z., C. F., and H. Z. revised the manuscript and approved the final version.

## Supporting information



Supporting Information

Supplemental Video 1

Supplemental Video 2

Supplemental Video 3

Supplemental Video 4

Supplemental Video 5

## Data Availability

The data that support the findings of this study are available from the corresponding author upon reasonable request.
