# Supplementary material for: Engineering Liquid Hierarchical Materials with DNA‐Programmed Spherical Nucleic Acids
Source: Adv Sci (Weinh). 2025 Jun 5;12(31):e04471. doi: 10.1002/advs.202504471 (PMC12376554; doi:10.1002/advs.202504471)
Supplement: Supplementary file 1 — Supporting Information [file ADVS-12-e04471-s006.docx]

**Engineering Liquid Hierarchical Materials with DNA-Programmed Spherical Nucleic Acids**

Zeyu Chen^1, 2 #^, Xu Chen^1, #^, Dan Lu^2^, Huating Kong^3^, Jingyi Ye^1^, Chunhai Fan^4^, Honglu Zhang^1, *^ and Huan Zhang^2, 4 *^

1, School of Sensing Science and Engineering, School of Electronic Information and Electrical Engineering, Shanghai Jiao Tong University, Shanghai 200240, China.

2, School of Agriculture and Biology, Shanghai Jiao Tong University, Shanghai 200240, China;

3, Shanghai Synchrotron Radiation Facility, Shanghai Advanced Research Institute, Chinese Academy of Sciences, Shanghai, 201204, China.

4, School of Chemistry and Chemical Engineering, New Cornerstone Science Laboratory, Frontiers Science Center for Transformative Molecules, National Center for Translational Medicine, Shanghai Jiao Tong University, Shanghai 200240, China

^#^Z. Chen and X. Chen contributed equally to this work. *Corresponding authors: Honglu Zhang, [zhanghonglu@scut.edu.cn](mailto:zhanghonglu@scut.edu.cn); Huan Zhang, [zhang_huan@sjtu.edu.cn](mailto:zhang_huan@sjtu.edu.cn)

**Materials and Reagents**

Sodium chloride, agarose, magnesium chloride, 50×TAE buffer, 5×TBE buffer, NTPs, DNase I, sucrose, 30% acrylamide, ammonium persulfate (APS), N, N, N', N'-Tetramethylethylenediamine (TEMED), DNA marker, 6×loading buffer and Tris (hydroxymethyl) aminomethane hydrochloride buffer (Tris-HCl, pH=8.0) were purchased from Sangon Bioengineering Technology and Services. The DNA oligonucleotides were purchased from and purified by Sangon Bioengineering Technology and Services; DNA oligonucleotides labelled with Cy3 or -SH were purified by HPLC, and all the oligonucleotides were dissolved in DNase/RNase-free water before use. The sequences are provided in Supplementary Table 1. Tris (2-carboxyethyl) phosphine (TCEP), n-butanol, and methanol were purchased from Shanghai Macklin Biochemical. Bis (sulfosuccinimidyl) phenylphosphine disodium salt (BSPP) was obtained from Sigma-Aldrich. The 5 nm, 10 nm and 15 nm AuNPs were purchased from Xi'an Ruixi Biological Technology. T7 RNA polymerase was obtained from Suzhou Novoprotein Scientific. mRNA was purchased from Genscript Biotech Corporation. T4 DNA ligase and Xfect™ RNA Transfection Reagent were procured from Takara Biomedical Technology (Beijing). HeLa cells were obtained from Procell Life Science & Technology. Fetal bovine serum, antibiotics, and culture media were all sourced from Thermo Fisher Scientific Inc. All other buffers and solutions were prepared using ultrapure water (>18.25 MΩ). All chemicals were used as received without further purification.

**Methods**

**Preparation of BSPP-protected AuNPs**

According to previous report (1), 3 mg BSPP was added to 10 mL of 15 nm AuNPs and shaken overnight at room temperature. After that, solid sodium chloride (NaCl) was slowly added to the mixture while stirring until the color changed from deep purple-red to light purple. The resulting mixture was centrifuged at 7000×g for 5 minutes to remove the supernatant. The AuNPs was then resuspended in 500 µL of 2.5 mM BSPP solution and mixed with 500 µL of methanol. The mixture was centrifuged again to remove the supernatant, and the product was resuspended in 2.5 mM BSPP solution.

**Synthesis of sgRNA**

sgRNA was obtained through *in vitro* transcription referring to previous reports (2), the DNA template was obtained by PCR method. The PCR thermal cycling program consisted of an initial denaturation step at 95°C for 5 minutes, followed by 30 cycles of denaturation at 94°C for 30 seconds, annealing at 44°C for 30 seconds, extension at 72°C for 30 seconds, and the final extension at 72°C for 10 minutes. The PCR product was stored at -20°C for subsequent use. *In vitro* RNA transcription was carried out by incubating 2 mM NTPs, 50 U T7 RNA polymerase, and 500 ng DNA template at 37°C for 4 h. DNase I was then added to digest the DNA template, and the RNA transcription product was purified by the Spin Column RNA Cleanup & Concentrator kit. The concentration of sgRNA was determined by Thermo NanoDrop One spectrophotometer.

**Synthesis of circular DNA**

The circular DNA was prepared according to a previously reported protocol (3). The template and primers were hybridized using the following PCR program to facilitate template circularization: 95°C for 2 minutes, 65°C for 2 minutes, 60°C for 6 minutes, followed by a step-wise decrease from 60°C to 20°C at a rate of -0.5°C per 30 seconds, totaling 80 cycles. The reaction was then held at 20°C for 30 seconds and finally cooled to 4°C for 10 minutes. 10× T4 DNA ligase buffer, 3U/μL T4 DNA ligase, and 10 μM circ-DNA precursor were added in a sample tube, placed in the PCR thermal cycler at 16°C for 1 hour, followed by heating to 65°C for 10 minutes. The nick of the cyclization product was closed by T4 DNA ligase, and obtained circ-DNA. The resulting PCR product was stored at -20°C for subsequent use.

**Preparation of SNAs**

DNA or RNA was added to citrate-modified AuNPs at the required molar ratio (Table S3) in the presence of 1 mM TCEP, resulting in the final volume of 10 μL. The above solution was mixed with 90 μL of n-butanol and rapidly vortexed for ~10 seconds. Subsequently, 10 μL of 0.5×TBE buffer (Tris, 44.5 mM; EDTA, 1 mM; boric acid, 44.5 mM; pH 8.0) was added to the solution, and a transient centrifugation was performed through a mini centrifuge to facilitate phase separation. Collecting the subaqueous solution from the immiscible liquid, which was the DNA/RNA-functionalized AuNPs, the particles were washed twice by centrifugation. In the end, the particles were resuspended in 0.5×TBE buffer. The entire process was carried out at room temperature and assisted by vortex mixing and centrifugation to promote phase separation. The time from solution mixing to product recovery was less than 1 minute. Different concentrations were used for different sizes of AuNPs (70 nM for 5 nm AuNPs, 30 nM for 10 nm AuNPs, and 15 nM for 15 nm AuNPs).

**Quantification of the DNA numbers on each SNAs**

The obtained SNAs samples were centrifuged at 21,000×g for 60 minutes for 5 nm AuNPs, 17,000×g for 60 minutes for 10 nm AuNPs, and 14,000×g for 15 minutes for 15 nm AuNPs. Then, repeated the centrifugation process three times, and collected the supernatant, which represented the unconnected DNA. The DNA concentration was quantified through a microspectrophotometer. The average DNA linking density was determined by SNAs samples prepared from three parallel experiments. The concentration of AuNPs was determined by Ultraviolet-Visible absorbance spectroscopy, and the size-dependent extinction coefficients were acquired from the purchase information. The following coefficients were used to determine the molar concentrations of 5 nm, 10 nm and 15 nm AuNPs: 1.10×10^7^ M^-1^ cm^-1^, 1.01×108 M^-1^ cm^-1^ and 3.67×108 M^-1^ cm^-1^, respectively.

**Gel electrophoresis**

The SNAs complexes were loaded onto a 2% agarose gel, and 2 μL of 50% sucrose was added to each sample. Electrophoresis was performed in 0.5×TBE buffer at the voltage of 60V in ice bath. Gel images were captured by a digital camera. Agarose gel electrophoresis and polyacrylamide gel electrophoresis (PAGE) experiments were conducted with the Bio-Rad electrophoresis system. Gel imaging analysis was performed by the Tanon-2500B gel imaging analysis system.

**Spectroscopic and other physical characterizations**

UV-Visible absorption data were acquired using a Hitachi UH-5700 spectrophotometer. The DNA concentration was measured by the Thermo NanoDrop One microspectrophotometer. TEM imaging was performed by JEM-1400Flash and Talos L120C G2 transmission electron microscope, both operated at 120 kV acceleration voltage. STEM imaging was performed by Thermo Scientific Talos F200X G2 transmission electron microscope, operated at 200 kV acceleration voltage. Dynamic light scattering measurements were conducted using the Malvern Nano-ZS instrument to determine the hydrodynamic size of the particles employed, measured at room temperature in aqueous solutions. Typically, 1 nM AuNPs or SNAs samples were dispersed in water for measurements. The excitation wavelength used was 633 nm, with a maximum laser output power of 10 mW. Centrifugation was carried out with Eppendorf centrifuge 5424R. PCR tests and isothermal incubation were performed with Bio-Rad T100 PCR thermal cycler.

**Cell culture and mRNA transfection**

HeLa cells were cultured in MEM medium containing 10% fetal bovine serum and antibiotics (100 μg/mL streptomycin and 100 μg/mL penicillin). The cells were cultured in a 5% CO_2_ incubator at 37°C.

The transfection procedure was conducted following the Takara Xfect™ RNA Transfection Reagent kit instructions. One day prior to transfection, plate cells in 1 ml of fresh growth medium containing 10% FBS so that the cells will be 80–90% confluent at the time of transfection. The Xfect RNA transfection polymer was thoroughly mixed before use. For each sample, two centrifuge tubes were prepared to dilute GFP expressing mRNA and transfection polymer separately with transfection buffer according to the kit's recommended ratios. The solutions were mixed, and incubated at room temperature for 10 minutes to form nanocomplexes. The entire nanocomplex solution or mRNA assembled SNAs was gently added to the cell culture medium, and the plate was gently rocked back and forth for better mixing. The plates were then incubated at 37°C in a cell incubator for 4 hours. Afterward, the transfection complexes were removed by aspirating the media and replaced with 1 mL fresh media. HeLa cells was observed under confocal laser scanning microscope after 24 hours post-transfection to check the expression of GFP.

**Confocal laser scanning microscope (CLSM) characterization**

mRNA expression was characterized using CLSM (Leica, TCS SP8 STED 3X). Excitation was set at 488 nm, and emission spectra were recorded between 500 and 600 nm. Characterization of Cy3-DNA droplets and Cy3-SNA droplets were performed with excitation at 561 nm, and emission spectra were recorded at 570-650 nm. Quantitative analysis of the mean fluorescence intensity of CLSM images was performed using Image J.

**Construction of hierarchical structures through SNAs assembly**

The 15 nm AuNPs was functionalized with SH-DNA or polyA-DNA, which were hybridized in 100 mM NaCl with excess 5 nm AuNPs (according to the DNA number ratio of 1:2). The mixture was incubated at 37°C for 2 hours and then annealed to room temperature overnight. The final concentration of 15 nm SNAs was 3 nM. Subsequently, the sample was centrifuged at 10,000 g for 10 minutes to remove the unhybridized 5 nm AuNPs. 10 μL of 1 nM hierarchical assembles sample solution was deposited onto the copper gird and air-dried at room temperature. Imaging of the sample was performed using the JEM-1400Flash transmission electron microscope at an accelerating voltage of 120 kV. The hybridization efficiency was assessed by counting the number of SNAs ligand about over 100 hierarchical assembles through TEM images, and the statistical analysis of data was performed using Gaussian distribution fitting by GraphPad software. The significant differences between different hybridization groups were analyzed using one-way analysis of variance by GraphPad software.

**Synthesis of three-branched and four-branched DNA** **nanostructures**

The DNA strands for dsDNA (Table S1, S1, S2), three-branched (Table S1, C1, C2-three-branched, and C4) and four-branched (Table S1, C1, C2, C3, and C4) DNA structures were mixed in a tube with a final concentration of 20 μM of each strand in a buffer consisting of 10 mM Tris-HCl. The sample was heated at 95°C for 5 min and then cooled down to 4°C in a PCR thermal cycler (Bio-Rad) to obtain dsDNA, three- and four-branched DNA nanostructures. For Cy3 labelled structures, C1 was replaced by C1-Cy3 accordingly.

**Construction of DNA droplets and SNA droplets**

DNA strands of dsDNA, three-branched and four-branched DNA structures were mixed and then denatured and annealed at a rate of −1°C/min to 25°C, using a thermocycler (Bio-Rad). All-DNA droplets are formed by mixing four-branched DNA nanostructures in 350 mM NaCl and incubating at 37°C for one hour, with a final concentration of 5 μM for each strand. To normalize the concentrations of 2-, 3-, and 4-branched DNA on the SNAs, we incubated SNAs (10 nm) at concentrations of 100, 66, and 50 nM, respectively, in 350 mM NaCl at 37°C with constant shaking for one hour. The DNA concentration in each SNAs system was maintained at 1 μM. To construct SNAs droplets, DNA monomers with SH, polyA and random sequence were first assembled onto AuNPs following the previous assembly steps to obtain SNAs with various valency. Subsequently, SNAs were annealed to form SNA droplets, with a final concentration of 300 nM and 50 nM for 5 nm and 10 nm AuNPs, respectively. SNA droplets formed by SH, polyA- and random sequence-SNAs were constructed at 350 mM, 100 mM and 100 mM NaCl, respectively.

**FRAP experiments**

After the formation of DNA droplets and SNA droplets, FRAP experiments were conducted using CLSM. Photobleaching was performed at 561 nm at 100% intensity for 20 seconds, causing complete disappearance of fluorescence in the target region. A series of images were obtained with a scan rate of one frame per minute, and the recovery time was monitored for approximately 16 minutes until the fluorescence intensity reached a plateau. The change of the average fluorescence intensity of the bleached region at various timepoint was recorded, through which we could draw the fluorescence recovery curve, and fitted with single exponential using Origin software.

**FRAP data analysis**

Based on previous studies (4), FRAP data analysis was constructed by the fluorescence recovery curve. The formula is as follows:

$$MF=\frac{I_{\infty}-I_{C}}{I_{C0}-I_{C}}$$

$$D_{app}=0.25\frac{\omega^{2}}{t_{1/2}}$$

The fluorescence recovery efficiency of droplets was calculated based on mobile fraction (MF), where I_∞_ is the final fluorescence intensity after recovery, I_C_ is the fluorescence intensity after photobleaching, and I_C0_ is the initial fluorescence intensity before photobleaching. The diffusion coefficient (D_app_) was calculated using the formula mentioned above, where ω is the radius of the bleached area, and t_1/2_ is the half-life value. The fluorescence recovery curve was fitted with single exponential by Origin software, where the t value in the fitting equation corresponds to half-life t_1/2_.

**Calculation of the density of valences within SNA droplets**


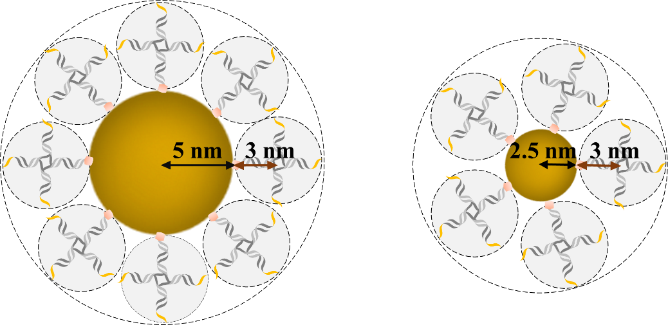


We define the density of valences as the number of sticky ends per unit volume of droplets. As shown in the above image, we model the four-branched DNA, 10 nm and 5 nm AuNPs as spheres with radii of 3 nm, 5 nm and 2.5 nm, respectively. Using the volume formula for a sphere, we determined the number of four-branched DNA conjugated on 5 and 10 nm SNA monomers to be approximately 11 and 22, respectively. It is noteworthy that these represent theoretical maximum numbers of four-branched DNA that could be conjugated on a spherical nanoparticle, taking into account steric hindrance and repulsion effects. Thus, the concentration of four-branched DNA used to construct DNA droplets, 5 nm and 10 nm SNA droplets is 5 μM, 3.3 μM and 1.1 μM, respectively. As one end of the four-branched DNA needed to conjugate with AuNPs, the number of sticky ends to form interactive DNA bonds on SNA monomers was determined to be 3. The number of sticky ends per unit volume was calculated as the ratio of the number of sticky ends to the volume of the AuNPs. For 5 nm and 10 nm SNAs, the ratios are 0.17 and 0.042, respectively. It is noteworthy that the 5 nm SNAs have almost four-fold higher density than the 10 nm SNAs.

**Fusion dynamics of SNA droplets**

To investigate the fusion dynamics of SNA and all-DNA droplets, we prepared 300 nM SNA droplets and 5 μM DNA droplets in a 350 mM NaCl buffer, respectively. The droplets were imaged at one-minute intervals using laser scanning confocal microscopy (LSCM). The aspect ratio *AR(t)* of the droplets was calculated for each image using the formula: *AR(t) =* *l_long_/l_short_*, where *l*_long_ and *l*_short_ represent the lengths of the droplet’s major and minor axes, respectively. Measurements were carried out using ImageJ software. The relaxation time *τ* was determined by fitting the aspect ratio data to the following exponential decay expression: *AR(t) = 1+(AR(0)-1) · exp(-τ/t).* The characteristic length scale *l* of the fusing condensates was defined as the geometric mean*: l = [(l_long_ (t = 0) − l_short_ (t = 0)) ∙ l_short_ (t = 0)]^1/2^*. The relationship between 𝜏 and 𝑙 follows the equation: *𝜏 = (μ/γ) · l + C*, where the slope of the linear fit yields the inverse capillary velocity *μ/γ*. Here, *μ* represents the viscosity, and *γ* represents the surface tension of the condensate.

**Small angle X-ray scattering (SAXS) experiments.**

The droplets were transferred to 1.0-mm quartz capillary tubes (Sinopharm Chemical Reagent Co., Ltd) for SAXS measurements. All SAXS experiments were performed at the BL16B1 and BL10U1 beamlines of Shanghai Synchrotron Radiation facility (SSRF) with X-rays at a wavelength of 1.24 Å (10 keV). The sample-to-detector distance was determined using cowhells as a standard. The 2D scattering patterns were collected by a detector, and the typical exposure time for the measured samples was set to 5 seconds. 1D scattering data were obtained by taking a radial average of the 2D scattering patterns using Fit2D software and generating profiles of scattering intensity as a function of scattering vector q: q = 4π sinθ/λ, where θ is half of the scattering angle, 2θ and λ is the X-ray wavelength. The model fitting of scattering curves was performed using SasView software.

**Cellular Uptake**

HeLa cells were seeded into confocal laser scanning microscope (CLSM) dishes and incubated overnight. Subsequently, pre-constructed Cy3-labeled SNA droplets (5 nm, final concentration of 50 nM) were added to the dishes and incubated for 8 hours. After three washes with 1×PBS, the cells were stained using CellMask™ plasma membrane stain and observed via CLSM.

**Uptake mechanism evaluation through endocytic inhibition**

HeLa cells were seeded into confocal dishes and incubated overnight. Cells were washed with 1×PBS and then cultured in media containing a dissolved quantity of small molecule inhibitor, chlorpromazine (10 μg/mL), methyl-β-cyclodextrin (10 mmol/L), or wortmannin (150 ng/mL) for 1 hour at 37 ºC, 5% CO_2_ (5). After this treatment, cells were washed with 1×PBS, and then pre-constructed Cy3-labeled SNA droplets (5 nm, final concentration of 50 nM) were added to the dishes and incubated for 8 hours. After three washes with 1×PBS, the cells were stained using CellMask™ plasma membrane stain and observed via CLSM.

**Photothermal Experiment**

In HeLa cells, 100 nM 5 nm AuNPs, SNA droplets or all-DNA droplets were added separately. The AuNPs, SNA droplets and all-DNA droplets were irradiated with a 561 nm laser for 20 minutes, and confocal images were captured throughout the process. The photothermal effect was characterized by measuring and statistically analyzing the changes in the aspect ratio of the cells before and after laser irradiation (N=20).

**Cell Viability Evaluation**

The viability of cells treated with or without laser was assessed by a cell counting kit-8 (CCK-8, Beyotime, China) according to the standard protocol. HeLa cells were seeded in a 96-well plate and treated with SNAs or SNA droplets (5 nm, both final concentration of 100 nM) and incubated for 8 hours. One group was left untreated, while another group was irradiated with a 561 nm laser for 20 minutes, with parallel experiments conducted in three times. And then 10% CCK-8 solution was added into the 96-well plate. After being incubated for 1 h, the samples were measured at 450 nm absorbance using a microplate reader (Agilent BioTek, USA).

**Table S1.** **Sequences of oligonucleotides used in this study.**

| **Name** | **Sequences (from 5’ to 3’)** |
| --- | --- |
| **SH-one binding site** | SH-AAAAAAAAAATACACGCATCCTTAG |
| **A10-15** | AAAAAAAAAATACACGCATCCTTAG |
| **A15-15** | AAAAAAAAAAAAAAATACACGCATCCTTAG |
| **A30-15** | AAAAAAAAAAAAAAAAAAAAAAAAAAAAAATACACGCATCCTTAG |
| **A50-15** | AAAAAAAAAAAAAAAAAAAAAAAAAAAAAAAAAAAAAAAAAAAAAAAAAATACACGCATCCTTAG |
| **A80-15** | AAAAAAAAAAAAAAAAAAAAAAAAAAAAAAAAAAAAAAAAAAAAAAAAAAAAAAAAAAAAAAAAAAAAAAAAAAAAAAAATACACGCATCCTTAG |
| **cDNA** | CTAAGGATGCGTGTA |
| **Random DNA-23 nt** | AACGCTCACCACTTGAACACCTC |
| **Random DNA-41 nt** | TCTGACGTAGTGTATGCACAGTGTAGTAAGGACCCTCGCAT |
| **Random DNA-63 nt** | CAGTTGAGACGAACAYYCCTAAGTCTGAAATTTATCACCCGCCATAGTAGACGTATCACCAGG |
| **Circ-template** | TATCCAGCATTAGCAACATTTCCGATTCCGCCATACCCACTTTTATTCCCAT |
| **Circ-primer** | GCTGGATAATGGGAAT |
| **sgDNA-Primer-F** | TAATACGACTCACTATAGGGCAACTAGAATGCAGTGAAAAGTTTTAGAGCTAGAAATAGCAAGTTAAA |
| **sgDNA-Primer-R** | GCACCGACTCGGTGCCACTTTTTCAAGTTGATAACGGACTAGCCTTATTTTAACTTGCTATTTCTAGC |
| **SH-two binding sites** | SH-AAAAAAAAAATACACGCATCCTTAGTACACGCATCCTTAG |
| **SH-three binding sites** | SH-AAAAAAAAAATACACGCATCCTTAGTACACGCATCCTTAGTACACGCATCCTTAG |
| **A30-three binding sites** | AAAAAAAAAAAAAAAAAAAAAAAAAAAAAATACACGCATCCTTAGTACACGCATCCTTAGTACACGCATCCTTAG |
| **SH-random** | SH-AAAAAAAAAACGTTGACACTGTCACGACATGTACCTGATATACACGCATCCTTAG |
| **SH-dsDNA** | SH-AAAAAAAAAAGATTCCTACGCACATTACACGCATCCTTAG |
| **A15-dsDNA** | AAAAAAAAAAAAAAAGATTCCTACGCACATTACACGCATCCTTAG |
| **A50-dsDNA** | AAAAAAAAAAAAAAAAAAAAAAAAAAAAAAAAAAAAAAAAAAAAAAAAAAGATTCCTACGCACATTACACGCATCCTTAG |
| **SH-dsDNA-three binding sites** | SH-AAAAAAAAAAGATTCCTACGCACATTACACGCATCCTTAGTACACGCATCCTTAGTACACGCATCCTTAG |
| **ds-cDNA** | ATGTGCGTAGGAATC |
| **SH-cDNA** | SH-AAAAAAAAAACTAAGGATGCGTGTA |
| **A30-cDNA** | AAAAAAAAAAAAAAAAAAAAAAAAAAAAAACTAAGGATGCGTGTA |
| **A50-cDNA** | AAAAAAAAAAAAAAAAAAAAAAAAAAAAAAAAAAAAAAAAAAAAAAAAAACTAAGGATGCGTGTA |
| **A80-cDNA** | AAAAAAAAAAAAAAAAAAAAAAAAAAAAAAAAAAAAAAAAAAAAAAAAAAAAAAAAAAAAAAAAAAAAAAAAAAAAAAAACTAAGGATGCGTGTA |
| **SH-ds-cDNA** | SH-AAAAAAAAAAGATTCCTACGCACATCTAAGGATGCGTGTA |
| **A30-ds-cDNA** | AAAAAAAAAAAAAAAAAAAAAAAAAAAAAAGATTCCTACGCACATCTAAGGATGCGTGTA |
| **A50-ds-cDNA** | AAAAAAAAAAAAAAAAAAAAAAAAAAAAAAAAAAAAAAAAAAAAAAAAAAGATTCCTACGCACATCTAAGGATGCGTGTA |
| **A30-random-cDNA** | AAAAAAAAAAAAAAAAAAAAAAAAAAAAAACTAAGGATGCGTGTATATCAGGTACATGTCGTGACAGTGTCAACG |
| **C1** | CGCGAGCAAACAAGTCTACGAATCAATTCCCGTTGGC |
| **C2** | CGCGGCCAACGGGAATTGAAATGAGAGGGTCTGTAATA |
| **C3** | CGCGTATTACAGACCCTCTCAAACCGAGCATATGTCAG |
| **C4** | CGCGCTGACATATGCTCGGAACGTAGACTTGTTTGCT |
| **C1-Cy3** | CGCGAGCAAACAAGTCTACGAATCAATTCCCGTTGGC-Cy3 |
| **SH-C4** | SH-aaaaaaaaaaCTGACATATGCTCGGAACGTAGACTTGTTTGCT |
| **A10-C4** | aaaaaaaaaaCTGACATATGCTCGGAACGTAGACTTGTTTGCT |
| **A30-C4** | aaaaaaaaaaaaaaaaaaaaaaaaaaaaaaCTGACATATGCTCGGAACGTAGACTTGTTTGCT |
| **N30-random-C4** | gtacttgatctttgggcctctgaaatgggaCTGACATATGCTCGGAACGTAGACTTGTTTGCT |
| **C2-three-branched** | CGCGGCCAACGGGAATTGAAACCGAGCATATGTCAG |
| **SH-S1** | GCGCCAGCTAGTAGTCAATGAAAAAAAAAA-SH |
| **A10-S1** | GCGCCAGCTAGTAGTCAATGAAAAAAAAAA |
| **A30-S1** | GCGCCAGCTAGTAGTCAATGAAAAAAAAAAAAAAAAAAAAAAAAAAAAAA |
| **S2** | CATTGACTACTAGCTG |

**Table S2. Dynamic light scattering (DLS) measurement of AuNPs and the DNA/RNA assembled SNAs.**

| **AuNPs type** | **5 nm AuNPs** | **PDI** | **10 nm AuNPs** | **PDI** | **15 nm AuNPs** | **PDI** |
| --- | --- | --- | --- | --- | --- | --- |
| **Citrate-SNAs** | 11.7 ± 0.2 nm | 0.152 | 14.1 ± 0.4 nm | 0.268 | 18.1 ± 0.4 nm | 0.196 |
| **SH-SNAs** | 17.6 ± 0.9 nm | 0.172 | 29.1 ± 0.9 nm | 0.225 | 30.7 ± 0.4 nm | 0.172 |
| **PolyA-SNAs** | 15.1 ± 0.7 nm | 0.185 | 20.0 ± 0.7 nm | 0.231 | 28.2 ± 0.3 nm | 0.184 |
| **SH-dsDNA-SNAs** | 22.5 ± 0.7 nm | 0.171 | 31.7 ± 0.3 nm | 0.121 | 27.4 ± 0.9 nm | 0.229 |
| **PolyA-dsDNA-SNAs** | 14.3 ± 0.3 nm | 0.184 | 24.4 ± 0.4 nm | 0.166 | 27.5 ± 0.6 nm | 0.286 |
| **Random DNA-23 nt-SNAs** | 14.7 ± 0.3 nm | 0.209 | 19.2 ± 0.3 nm | 0.163 | 27.2 ± 0.9 nm | 0.277 |
| **Random DNA-41 nt-SNAs** | 12.3 ± 0.9 nm | 0.197 | 17.2 ± 0.5 nm | 0.255 | 29.7 ± 0.7 nm | 0.250 |
| **Random DNA-63 nt-SNAs** | 13.3 ± 0.9 nm | 0.228 | 20.0 ± 0.7 nm | 0.274 | 29.2 ± 0.3 nm | 0.263 |
| **Circ-DNA-SNAs** | 12.1 ± 0.7 nm | 0.273 | 20.3 ± 0.6 nm | 0.276 | 24.1 ± 0.2 nm | 0.250 |
| **SgRNA-SNAs** | 15.3 ± 0.8 nm | 0.246 | 20.5 ± 0.1 nm | 0.278 | 28.0 ± 0.8 nm | 0.259 |
| **mRNA-SNAs** | 15.6 ± 0.6 nm | 0.269 | 29.5 ± 0.2 nm | 0.263 | 32.6 ± 0.5 nm | 0.266 |

**Table S3. Assembly parameters of DNA and RNA onto AuNPs via the butanol dehydration method.**

| **AuNPs type** | | **5 nm AuNPs** | **10 nm AuNPs** | **15 nm AuNPs** |
| --- | --- | --- | --- | --- |
| **Concentration** | | 70 nM | 30 nM | 15 nM |
| **Centrifugation speed (×1000g)** | | 21 | 17 | 14 |
| **Molar ratio** | **AuNPs:SH-DNA** | 1:50 | 1:100 | 1:200 |
|  | **AuNPs:SH-four-branched DNA** | 1:100 | 1:200 | 1:500 |
|  | **AuNPs:PolyA-ssDNA** | 1:100 | 1:200 | 1:500 |
|  | **AuNPs:PolyA-dsDNA** | 1:100 | 1:200 | 1:500 |
|  | **AuNPs:PolyA-four-branched DNA** | 1:100 | 1:200 | 1:500 |
|  | **AuNPs:mRNA (contains polyA)** | 1:100 | 1:200 | 1:500 |
|  | **AuNPs:Random DNA-23 nt** | 1:200 | 1:500 | 1:1000 |
|  | **AuNPs:Random DNA-41 nt** | 1:300 | 1:750 | 1:1500 |
|  | **AuNPs:Random DNA-63 nt** | 1:300 | 1:1000 | 1:2000 |
|  | **AuNPs:Circ-DNA** | 1:300 | 1:1000 | 1:2000 |
|  | **AuNPs:SgRNA** | 1:300 | 1:1000 | 1:2000 |

**Supplementary text:**

Relative affinity calculations

The relative affinity of nucleic acids (NAs) for AuNPs is defined as the minimum AuNPs: NA ratio required to form stable SNAs. As reported in previous studies (6), a lower ratio indicates a higher affinity for AuNPs, and vice versa. Using the butanol dehydration method, we determined the lowest AuNPs: NA ratios needed to achieve stable SNA formation, as listed in Table S3. Based on these ratios, we generated a heatmap (Figure 1b) that visually represents the relative affinity of representative nucleic acids, quantified by their stoichiometric ratios to AuNPs.

**Table S4. The summary of mean number of SNAs ligand on each SNAs core programmed by DNA bonds.**

| **AuNPs type** | **5 nm AuNPs (ligand)** | | | | | | | |
| --- | --- | --- | --- | --- | --- | --- | --- | --- |
| **15 nm AuNPs (core)** | **Mean number of SNAs ligand** | A30-ssDNA | A50-ssDNA | A80-ssDNA | SH-ssDNA | SH-dsDNA | A30-dsDNA | A50-dsDNA |
|  | SH-one binding site | 1 | 1 | 1 |  | | | |
|  | SH-two binding sites | 4 | 3 | 4 |  |  |  |  |
|  | SH-three binding sites | 3 | 7 | 6 |  |  |  |  |
|  | SH-random (45 nt) | 2 |  | |  |  |  |  |
|  | A30-three binding sites | 6 | 6 | 7 |  |  |  |  |
|  | SH-dsDNA | 3 | 9 | 10 | 2 | 2 | 7 | **12** |
|  | A15-dsDNA | 3 | 8 | 6 | 2 | 2 | 4 | 7 |
|  | A50-dsDNA | 6 | 3 | 2 | 2 | 3 | 4 | 3 |
|  | SH-dsDNA-three binding sites |  | | | | 2 | **11** | **13** |

**Table S5. The inverse capillary velocities of all-DNA, all-RNA and SNA condensates droplets.**

| Droplets Type | Inverse Capillary Velocities (min/μm) | Number of Sticky Ends | Length of Sticky Ends | Reaction Buffer | References |
| --- | --- | --- | --- | --- | --- |
| All-DNA | 0.015 | three | 4 nt | 350 mM  NaCl | (7) |
| All-DNA | 0.36 | three | 6 nt | 200 mM  NaCl | (8) |
| All-DNA | 19.5 | four | 6 nt | 250 mM  NaCl | (9) |
| All-RNA | 2.12 | four | 6 nt | 10 mM MgCl_2_ | (10) |
| All-RNA | 57.81 | four | 6 nt | 500 mM NaCl 100 mM KCl | (11) |
| All-DNA | 2.07 | four | 4 nt | 350 mM  NaCl | this work |
| SNA | 10.26 | three | 4 nt | 350 mM  NaCl | this work |

**Supporting Figures**

**
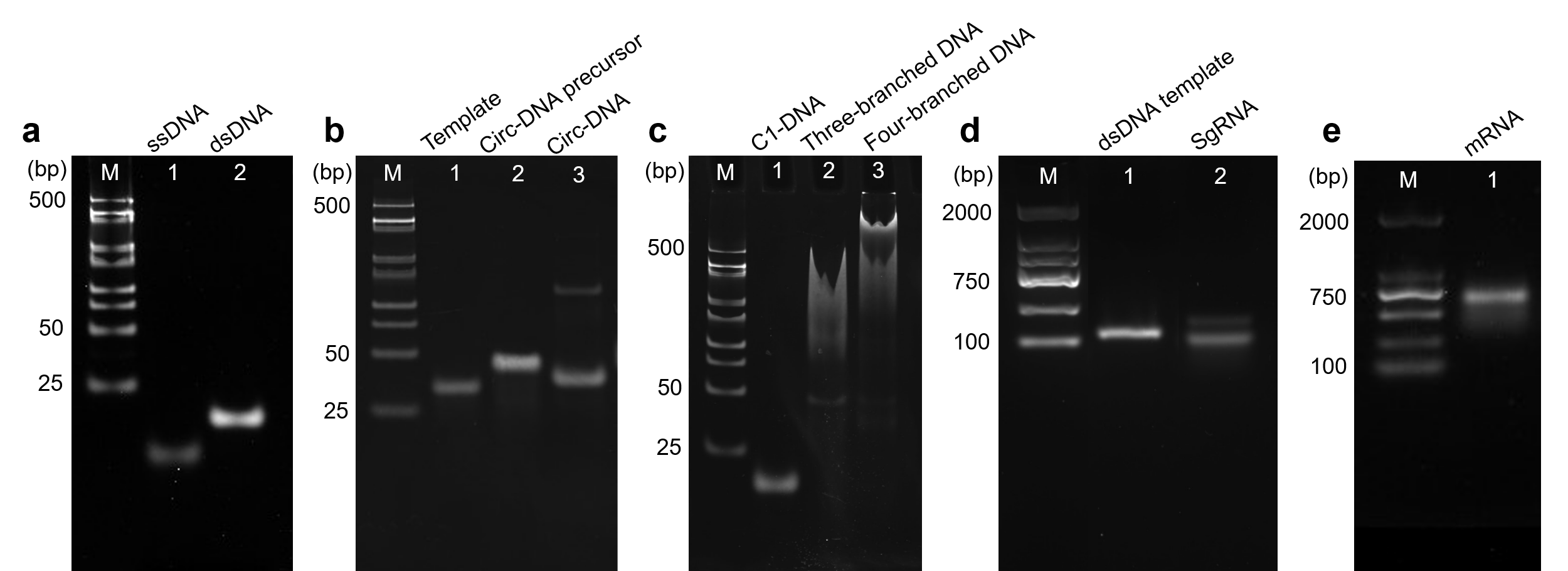
**

**Figure S1.** Gel electrophoresis analysis was carried out to characterize the DNA and RNA employed in this study. In order to characterize the double-stranded DNA, circular DNA, three-branched DNA and four-branched DNA, 10% PAGE was utilized, while 1% agarose gel electrophoresis was used to analyze the *in vitro* transcribed sgRNA and mRNA. M: marker. (a) PolyA10-ssDNA was loaded in Lane 1, and polyA10-dsDNA was loaded in Lane 2. (b) Lane 1 represented 52 nt DNA template, Lane 2 and Lane 3 represented the circular DNA precursor and circular DNA (the lower band). (c) Lane 1 represented the representative C1-ssDNA used for structure formation, lanes 2 and 3 represent three- and four-branched DNA. (d) Lane 1 was the double-stranded DNA template used for transcription, and Lane 2 was the obtained sgRNA (96 nt, the light higher band might be caused by the secondary structures of sgRNA). (e) Lane 1 was the mRNA used for GFP expression.

**
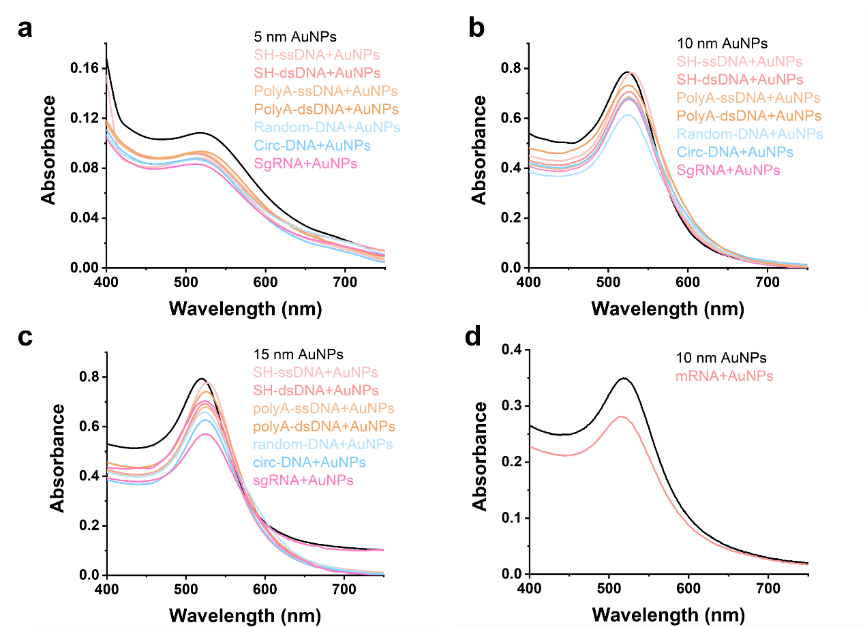
**

**Figure S2.** (a-d) The UV-Vis absorption spectra of different-sized AuNPs (5, 10, and 15 nm) before and after binding with different types of DNA or RNA, including thiol-modified DNA, polyA-tailed DNA, double-stranded DNA, random sequence DNA, circular DNA, sgRNA and mRNA. A redshift of 3, 5, and 7 nm, respectively, was observed in the absorption peaks of AuNPs compared to citrate-modified AuNPs, indicating the successful attachment of DNA or RNA onto the surface of AuNPs to form SNAs.

**
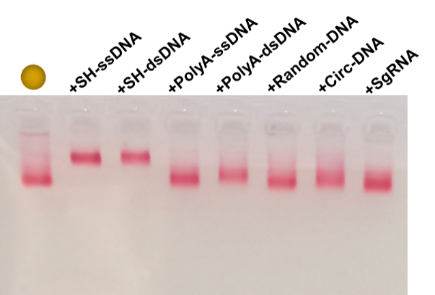
**

**Figure S3.** Gel electrophoresis of SNAs formed by 15 nm AuNPs with various types of nucleic acids. Lane 1 represents BSPP-capped AuNPs. The utilized DNA includes SH-one binding site, A15-15, random DNA-23 nt, Circ-DNA, and SgRNA.

**
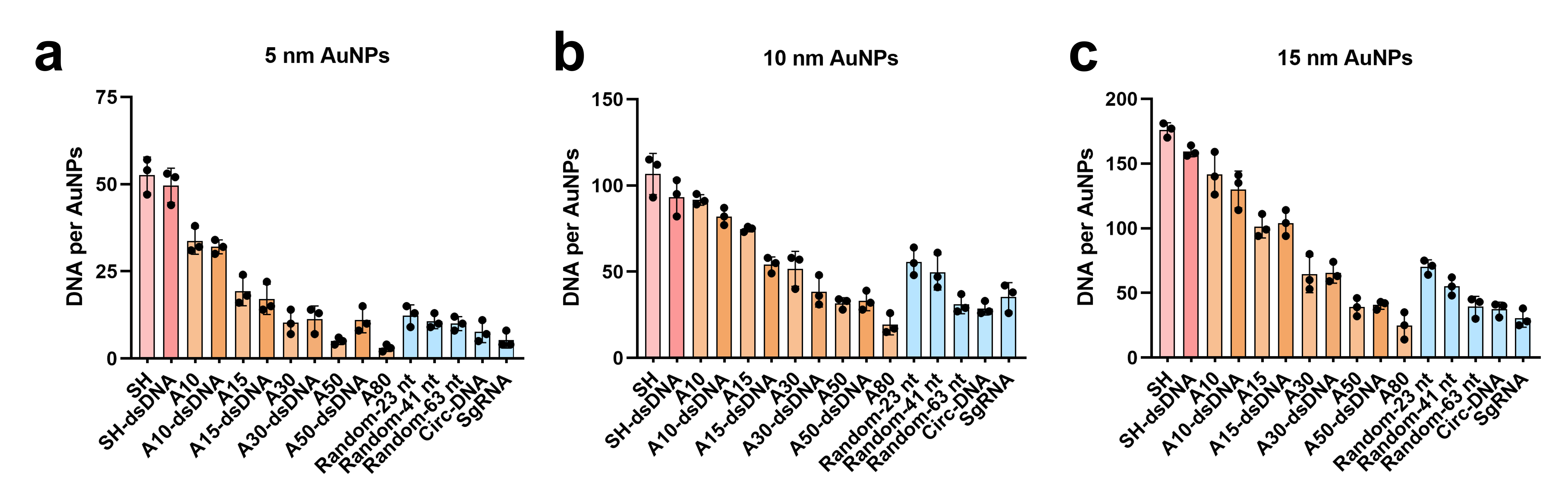
**

**Figure S4.** (a-c) The number of DNA or RNA probes on different-sized AuNPs (namely 5, 10, and 15 nm AuNPs) was quantified, and the sequences could be found in Table S1. The error bars indicated the standard deviation of three parallel experiments. Among them, thiol-modified DNA exhibited the highest assembly density, attributed to the formation of stable Au-S chemical bonds between thiol groups and AuNPs. The assembly density of polyA-tailed DNA decreased with an increasing number of adenine groups, which allowed to modulate the DNA density on AuNPs by assembling DNA with different polyA lengths. Moreover, the assembly density of double-stranded DNA closely resembled that of single-stranded DNA. The assembly density of random sequence DNA was negatively correlated with DNA chain length, and the assembly density was lower than that of polyA-tailed DNA.


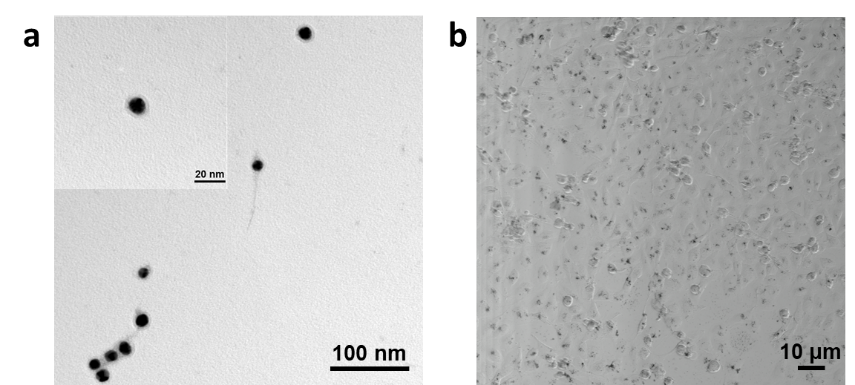


**Figure S5.** Representative TEM images of mRNA assembled SNAs, in which mRNA was stained with 2% uranyl acetate for 1 minute. Scale bars: 100 nm and 20 nm (insert image).


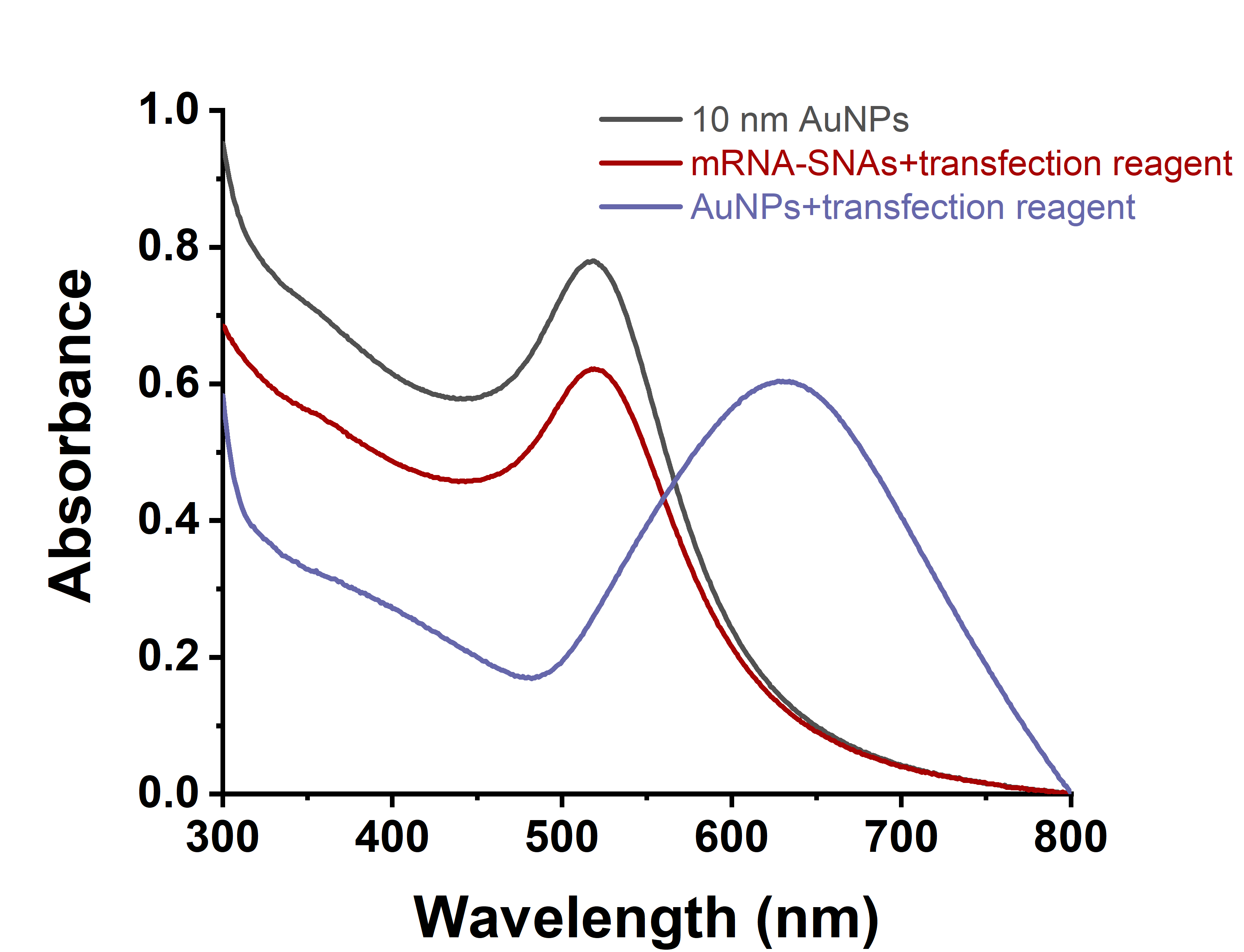


**Figure S6.** The UV-Vis absorption spectra of AuNPs (10 nm, black), AuNPs (blue) and mRNA-assembled SNAs (red) wrapped by transfection reagent. The results indicate that while AuNPs alone exhibit significant peak broadening and red shift (~110 nm) in the presence of the transfection reagent, suggesting aggregation and instability, the mRNA-assembled SNAs retain a stable spectrum, demonstrating their high colloidal stability and structural integrity under identical conditions.


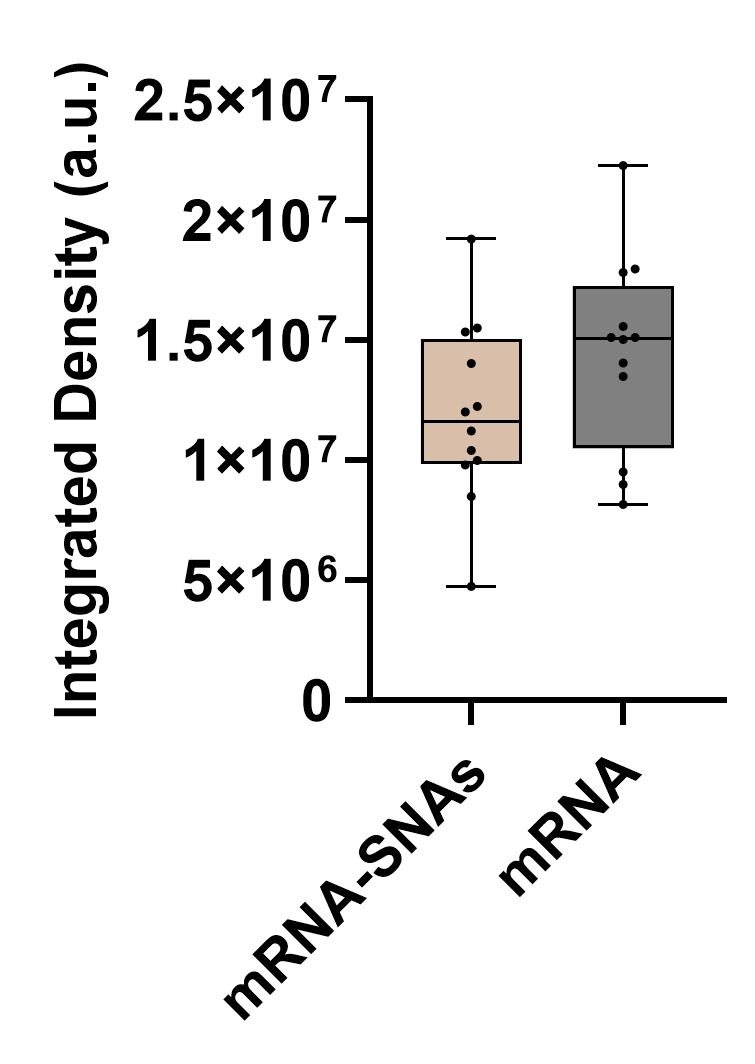


**Figure S7.** Analysis of fluorescence intensity of GFP expression following transfection with mRNA or mRNA-SNAs.


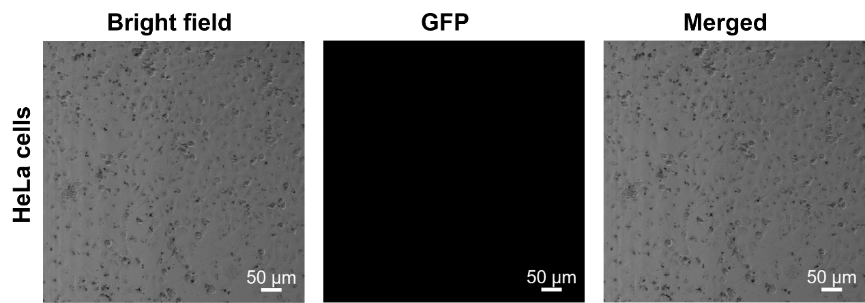


**Figure S8.** CLSM images of HeLa cells showing no express of GFP treated with mRNA assembled SNAs without the assistance of transfection reagent. Scale bars: 50 μm.

**
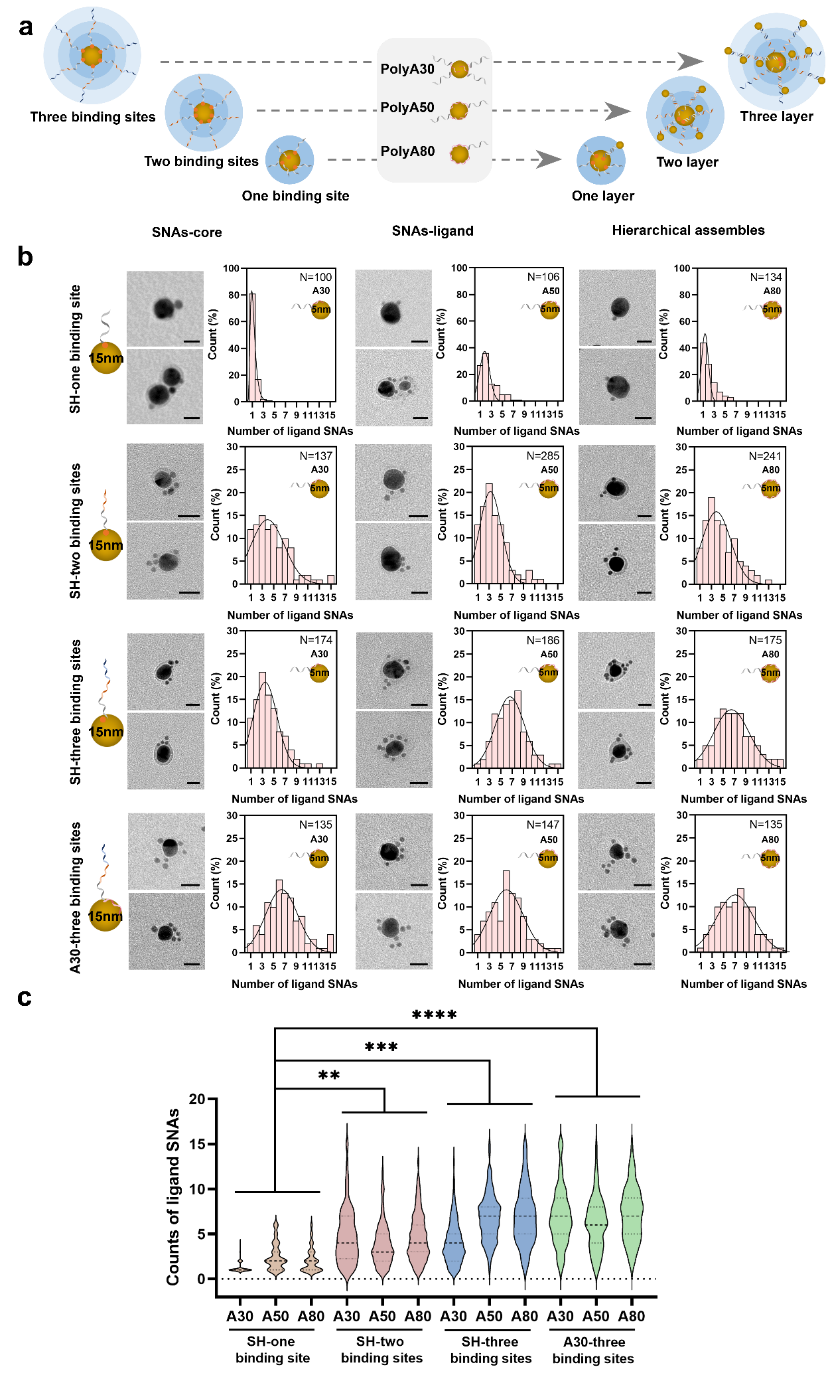
**

**Figure S9.** (a) Schematic representation of the longitudinal spatial configuration of DNA bonds on SNAs core (15 nm) and the lateral configuration of DNA bonds on SNAs ligand (5 nm) affecting the efficiency of hierarchical assembles. (b) Representative TEM images of hierarchical assembles formed by SNAs core (SH-one binding site, SH-two binding sites, SH-three binding sites and A30-three binding sites) with different polyA (polyA30, polyA50, and polyA80) regulated SNAs ligand. The distribution of the number of ligands bound to hierarchical assembles (N>100) is shown, with the black curve representing Gaussian distribution fitting. Scale bars: 20 nm. (c) Analysis of significant differences in the distribution of the number of ligands bound in different groups of hierarchical assembles. ****, P < 0.0001; **, P < 0.01, one-way analysis of variance.

**
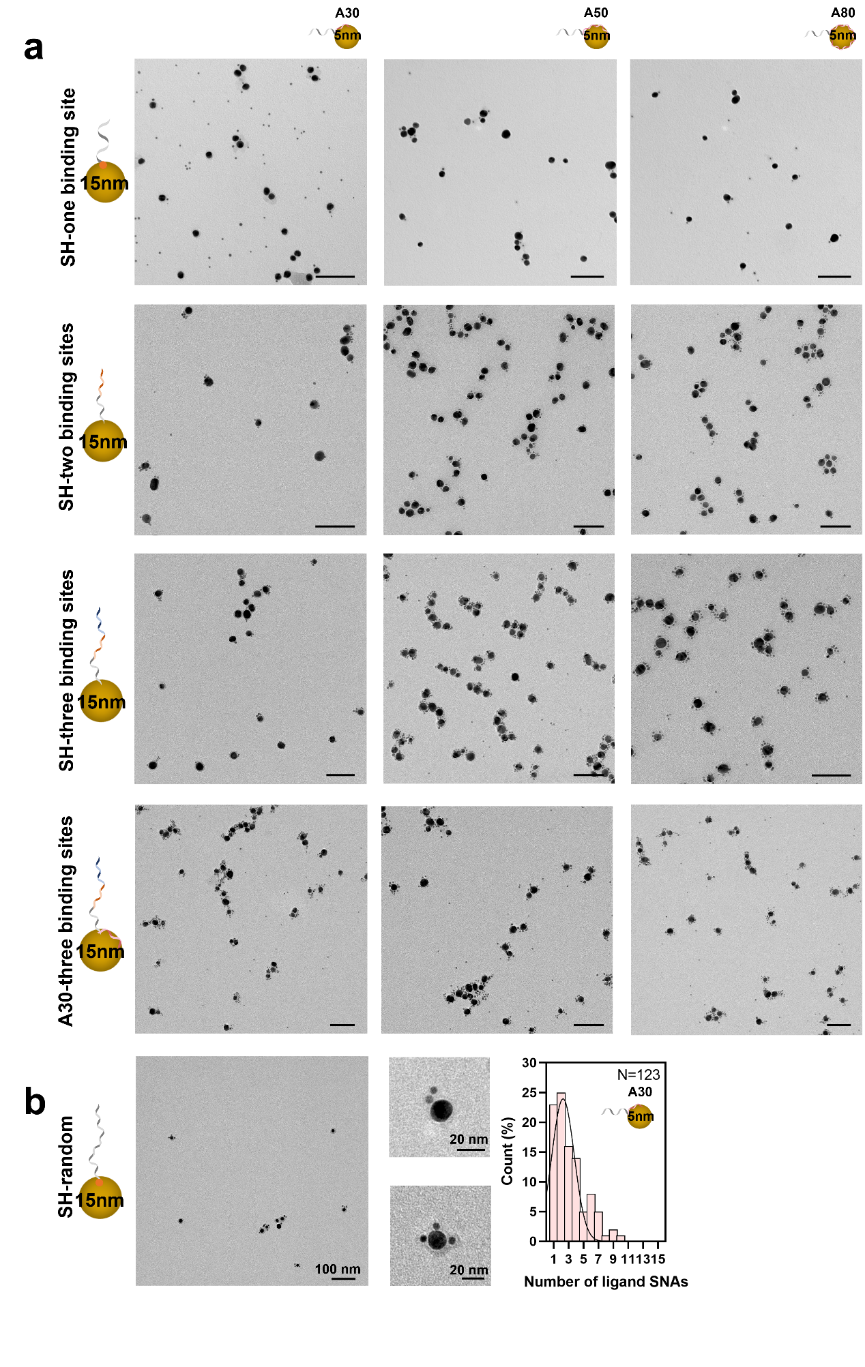
**

**Figure S10.** (a) Representative wide-field TEM images of hierarchical assembles formed by SNAs core (SH-one binding site, SH-two binding sites, SH-three binding sites and A30-three binding sites) and SNAs ligand regulated by different polyA (polyA30, polyA50, and polyA80). Scale bars: 100 nm. (b) Representative wide-field TEM images of hierarchical assembles formed by SH-random SNAs core and polyA30 ligand. Scale bars: 100 or 20 nm. The distribution of the number of ligands bound to each core (N>100) is illustrated, with the black curve representing the Gaussian distribution fitting.

**
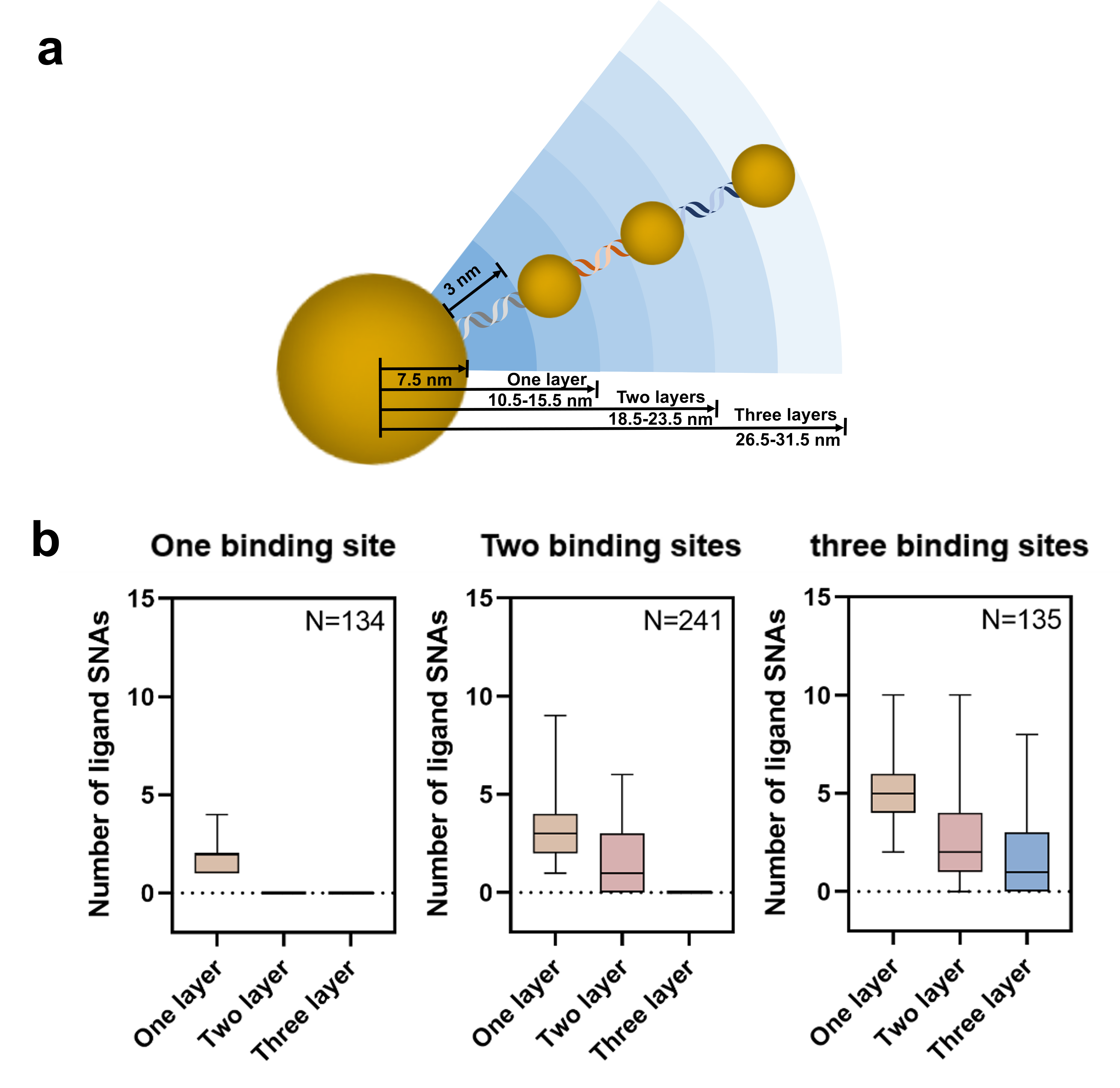
**

**Figure S11.** (a) Definition of interlayer distances for multi-site bound ligands on SNAs, showing regions corresponding to the first (10.5-15.5 nm), second (18.5-23.5 nm), and third layers (26.5-31.5 nm). (b) Statistical analysis showing the distribution of multi-site bound ligands across different layers, highlighting the differences observed with an increasing number of binding sites.

**Supplementary text:**

Based on previously reported study (12), the observed hybridization distances between SNAs under electron microscopy are generally shorter than the theoretical DNA bond length, which could be attributed to factors such as drying effects or the distribution of duplexes DNA bonds may not be perfectly perpendicular to the AuNPs surface. By referencing this study and considering of the hybridization sequence lengths (15 bp, approximately 3 nm under TEM image) we used here, we defined the first layer as the region where the distance from the centers of the 15 nm SNAs core to the 5 nm ligand is 10.5-15.5 nm, and correspondingly the second layer is 18.5-23.5 nm, and the third layer is 26.5-31.5 nm.

**
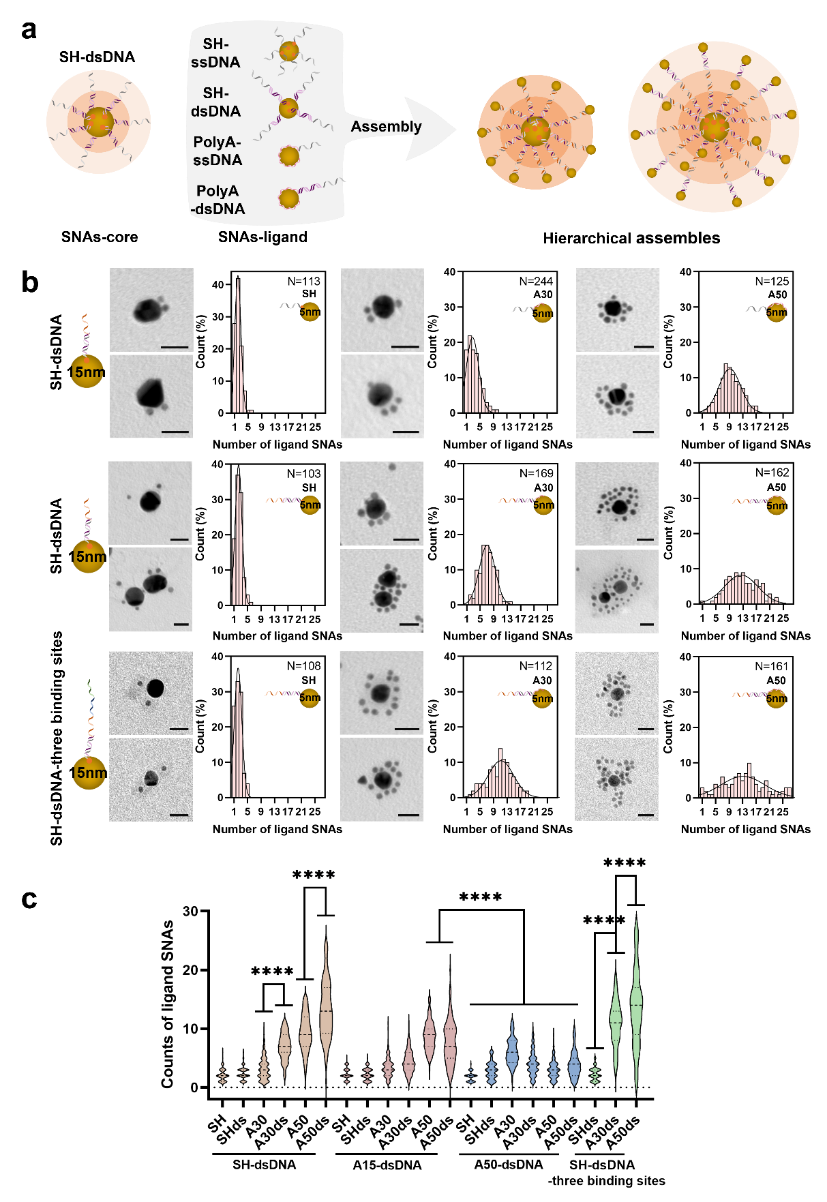
**

**Figure S12.** (a) Schematic diagram illustrating the influence of the rigid dsDNA structure of DNA bonds on the SNAs core or ligand on the efficiency of hierarchical assembles formation. (b) Representative TEM images of hierarchical structures assembled by SNAs core with dsDNA structure DNA bonds (SH-dsDNA and SH-dsDNA-three binding sites) and SNAs ligand with different DNA bonds regulation (SH, polyA30, polyA50, SH-dsDNA, polyA30-dsDNA, polyA50-dsDNA). The distribution of the number of ligands bound to each hierarchical assembles (N>100) is shown with the black curve representing a Gaussian distribution fit. Scale bars: 20 nm. (c) Analysis of significant differences in the distribution of the number of ligands bound in different groups of hierarchical assembles. ****, P < 0.0001, one-way analysis of variance.

**
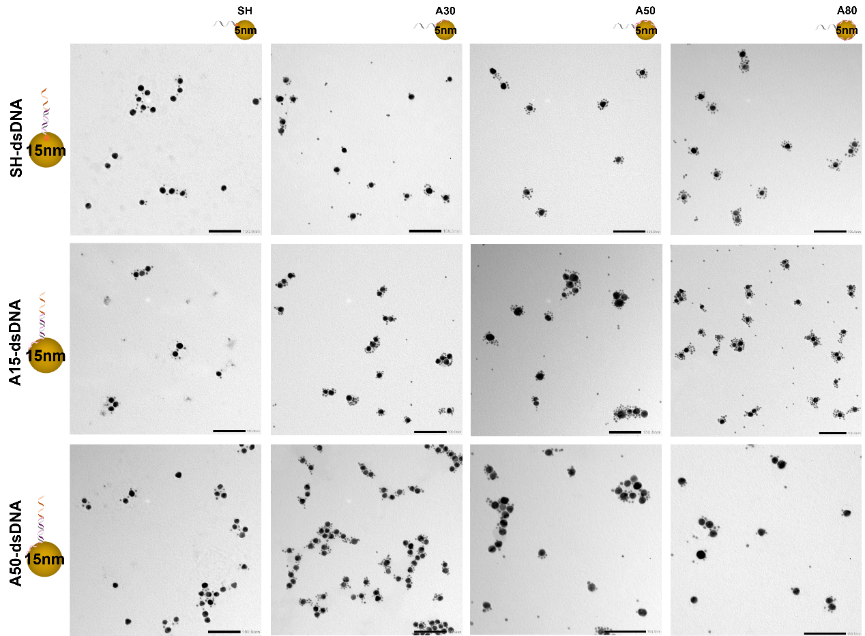
**

**Figure S13.** The representative wide-field TEM images of hierarchical assembles formed by SNAs core with rigid dsDNA region (SH-dsDNA, polyA15-dsDNA and polyA50-dsDNA) and SNAs ligand with different density of DNA bonds (SH, polyA30, polyA50, and polyA80). Scale bars: 100 nm.

**
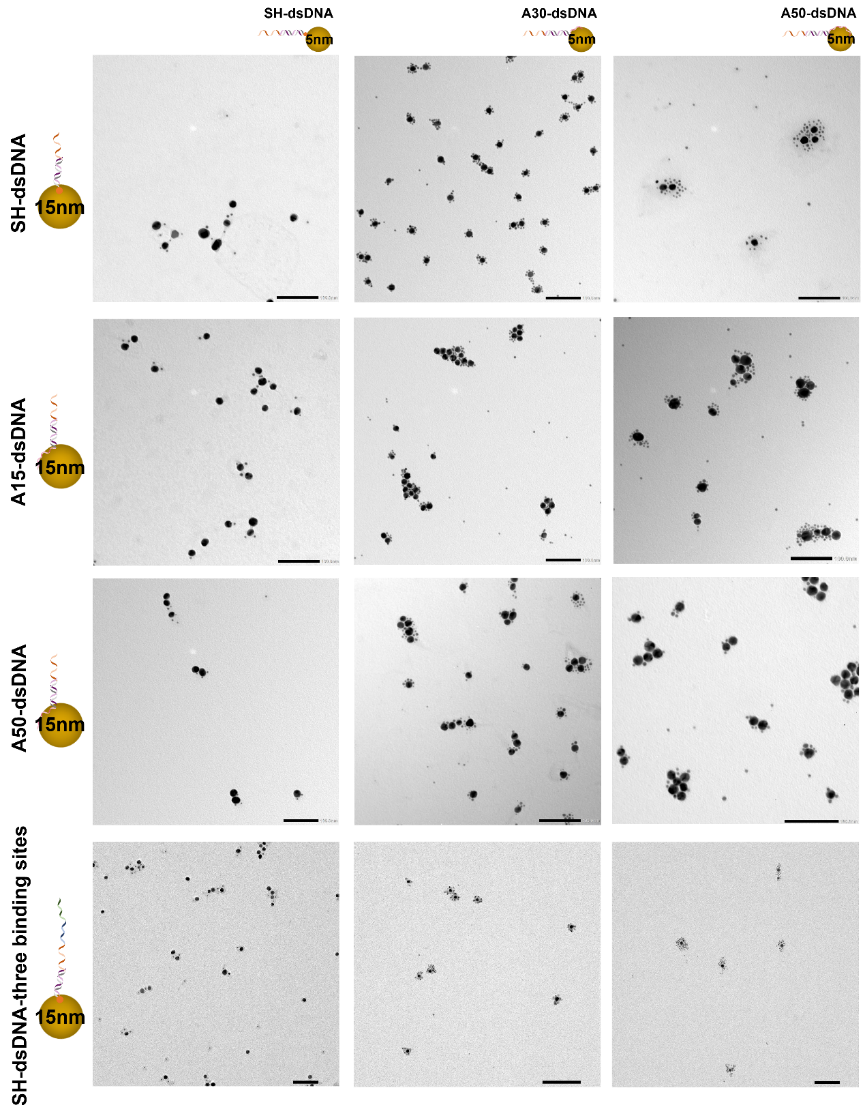
**

**Figure S14.** Representative wide-field TEM images of hierarchical assembles formed by SNAs core (SH-dsDNA, polyA15-dsDNA, polyA50-dsDNA and SH-dsDNA-three binding sites) and SNAs ligand (SH-dsDNA, polyA30-dsDNA and polyA50-dsDNA). Scale bars: 100 nm.

**
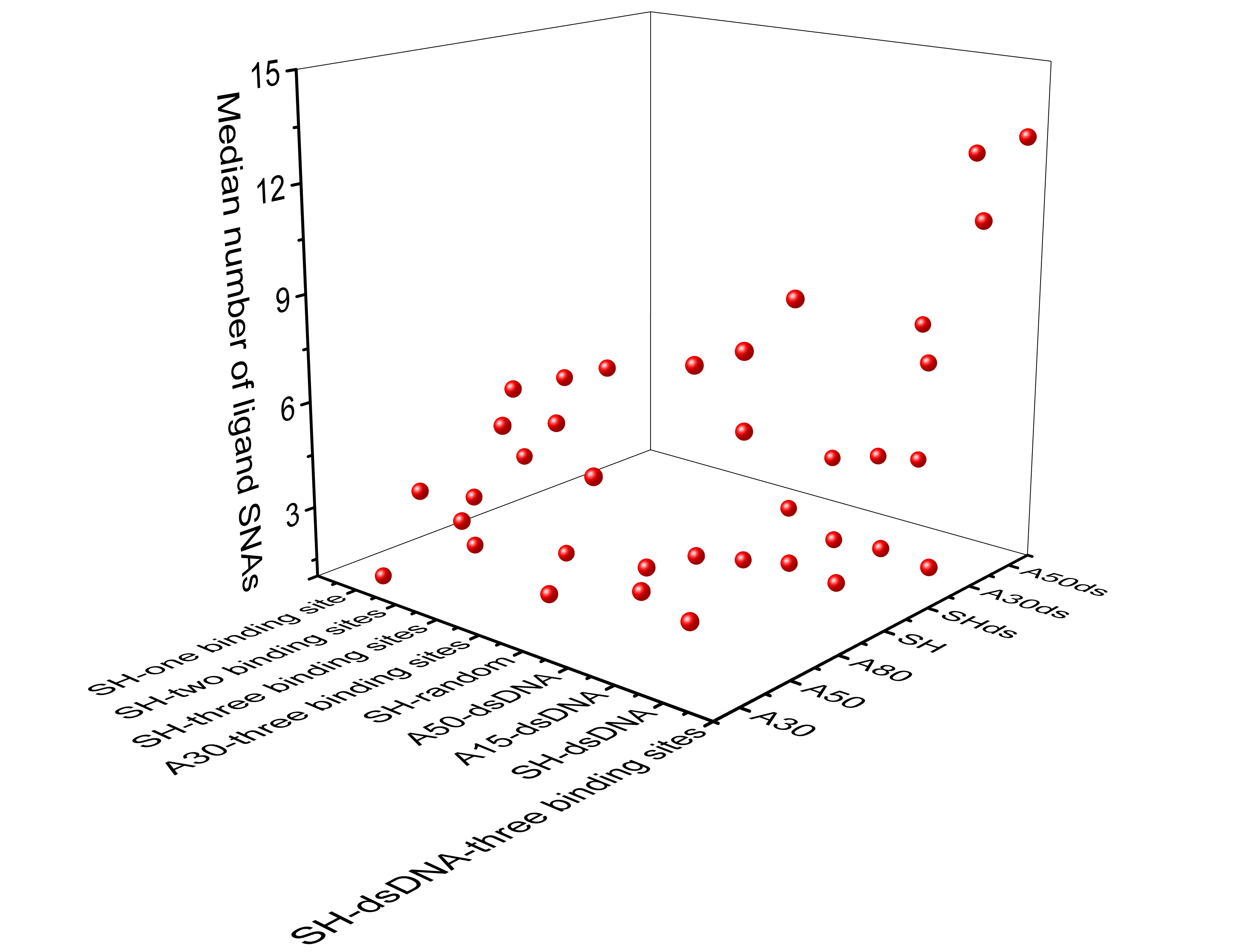
**

**Figure S15.** The summary of median number of ligands bound on each core under different core and ligand conditions.

**Supplementary text:**

We found the use of polyA to reduce spatial hindrance further improved assembly efficiency. For instance, SNA cores with three binding sites and ligands constructed with longer polyA sequences (polyA50 and polyA80) demonstrated a two-fold increase in assembly efficiency compared to polyA30 (Figure S7c, Table S4). To investigate the synergistic effects of multiple binding sites and polyA regulation, we designed a polyA30-SNA core with three binding sites (Figures S7b and S7c), which showed a six-fold increase in assembly compared to SNAs cores with a single binding site (Table S4).

Furthermore, we observed that the assembly efficiency of SH-A10 SNAs-core or -ligand with either ssDNA or dsDNA bond did not show significant difference and the assembly efficiency is always low. This result indicates that an excessive DNA bond density on SNAs (both core and ligand) is not favorable for further assembly (Table S4 and Figure S4). However, when we applying the polyA enabled regulating strategy together with the dsDNA rigid bond when constructing SNAs core, the number of 5nm SNAs bound to SNAs-core is lower than that to SH-dsDNA and A15-dsDNA constructed SNAs core (Figure S10 and Table S4). We attribute this to the much lower number of DNA bond (42 VS 160 and 104) on SNAs-core, which plays the defining role during the assembly (Figure S4).

**
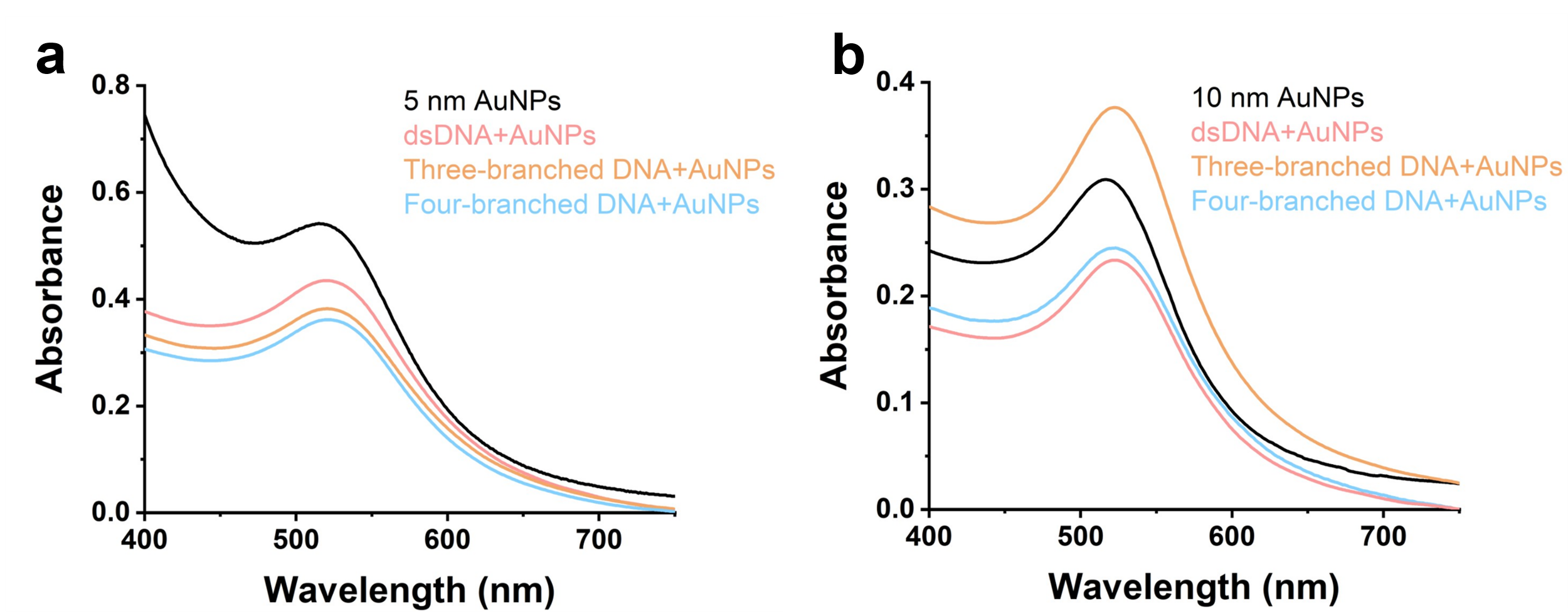
**

**Figure S16.** The UV-Vis absorption spectra of 5 nm (a) and 10 nm (b) AuNPs assembled by dsDNA, three- and four-branched DNA.

**
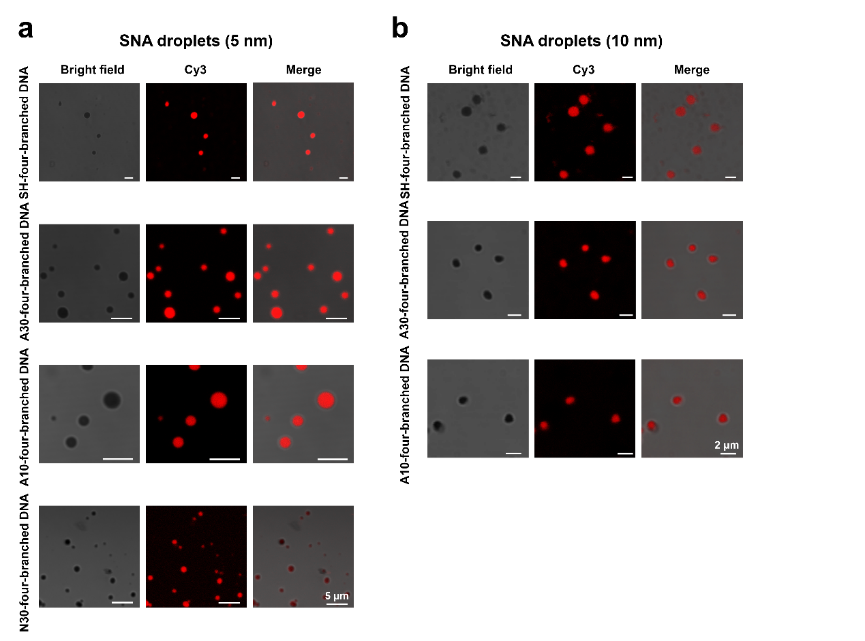
**

**Figure S17.** The representative CLSM images of SNA droplets formed by (a) 5 nm SNAs (SH-, polyA30-, polyA10- and N30-random-four-branched DNA) and (b) 10 nm SNAs (SH-, polyA30- and polyA10-four-branched DNA). Scale bars: 5 μm and 2 μm.

**
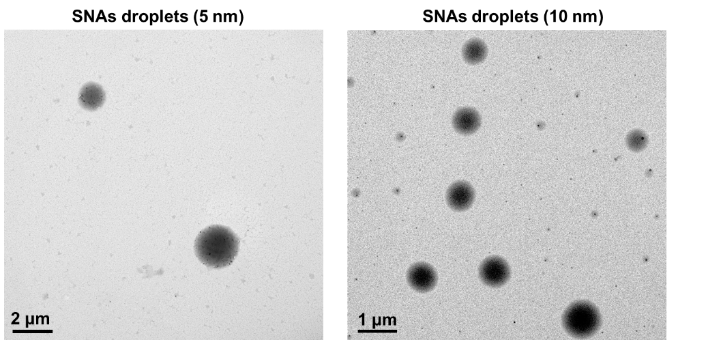
**

**Figure S18.** TEM images demonstrated the formation of SNA droplets (5 and 10 nm).

**
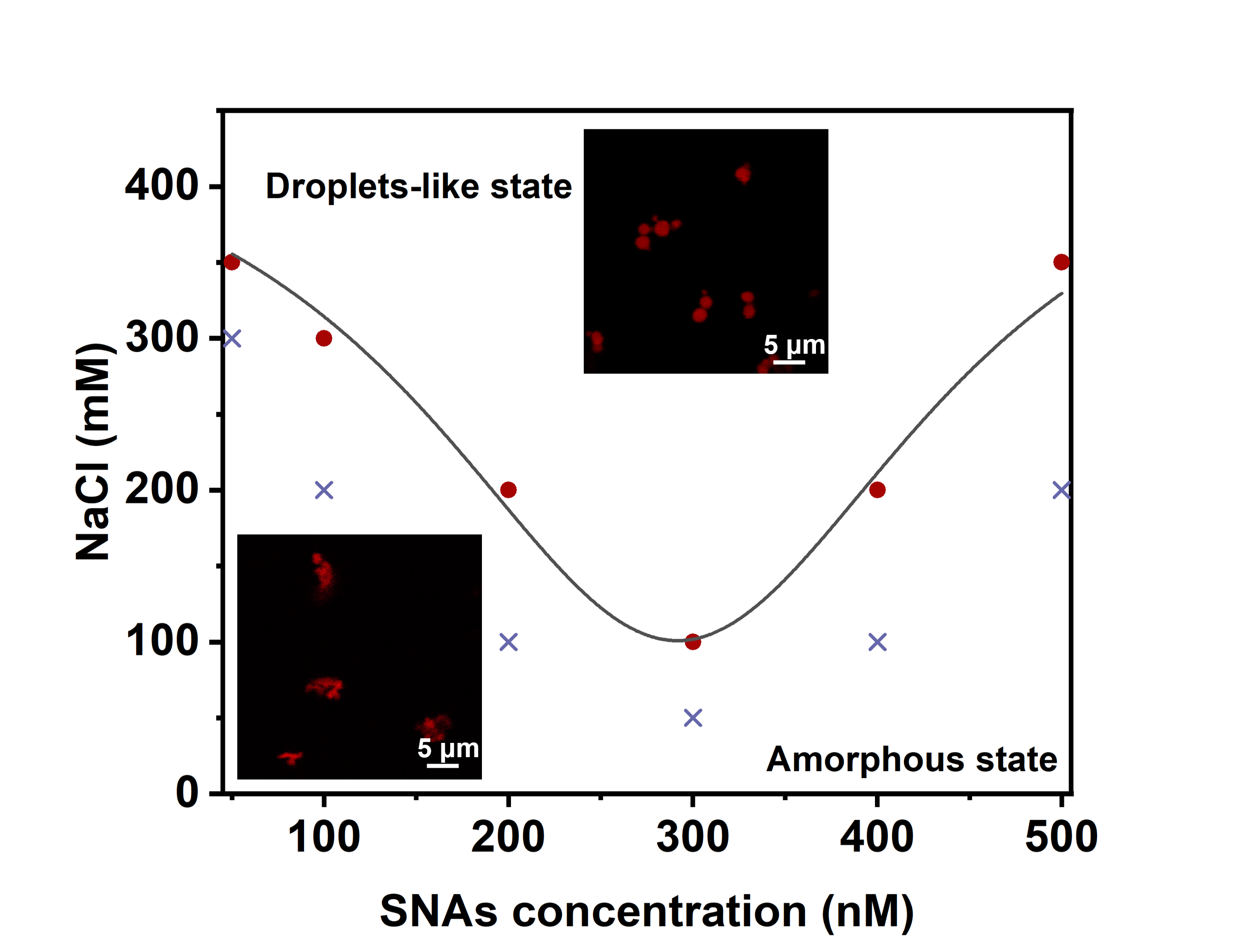
**

**Figure S19.** Phase diagram of the SNAs at different salt concentrations. Red circles indicate the phase separation boundary, i.e., the formation of well-rounded and dispersed SNA droplets. Blue crosses mark conditions that result in amorphous condensates. The black line represents the fitted phase separation curve based on the data points. The region above the curve indicates the droplets-like state, while the region below it corresponds conditions leading to dispersed or amorphous structures. Inserts display representative CLSM images of the droplets-like state and amorphous condensates. Scale bars: 5 μm.

**
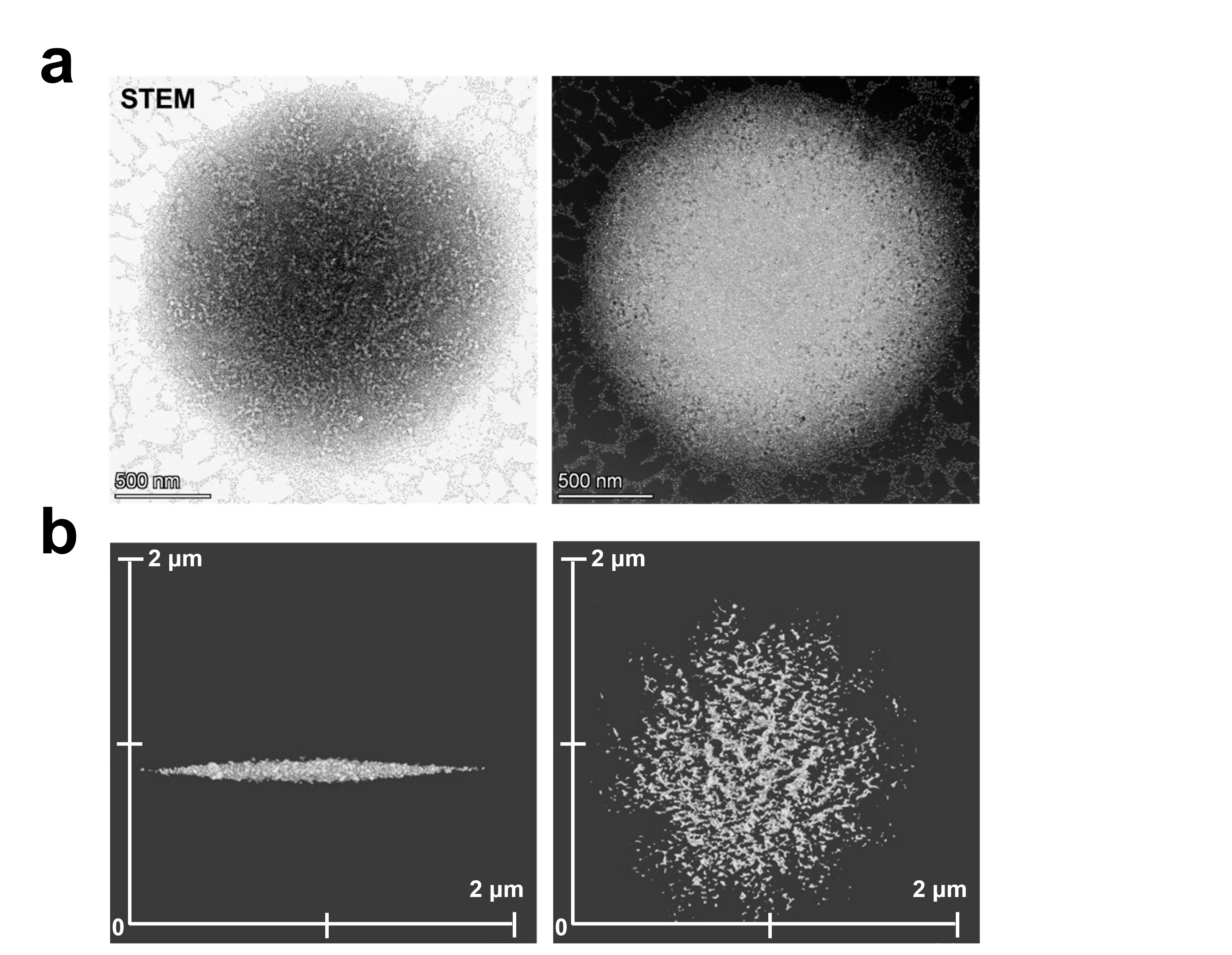
**

**Figure S20.** (a) The BF and HAADF STEM image of SNA droplets (5 nm). Scale bars: 500 nm. (b) The STEM reconstruction tomography images of SNA droplets (5 nm).

**
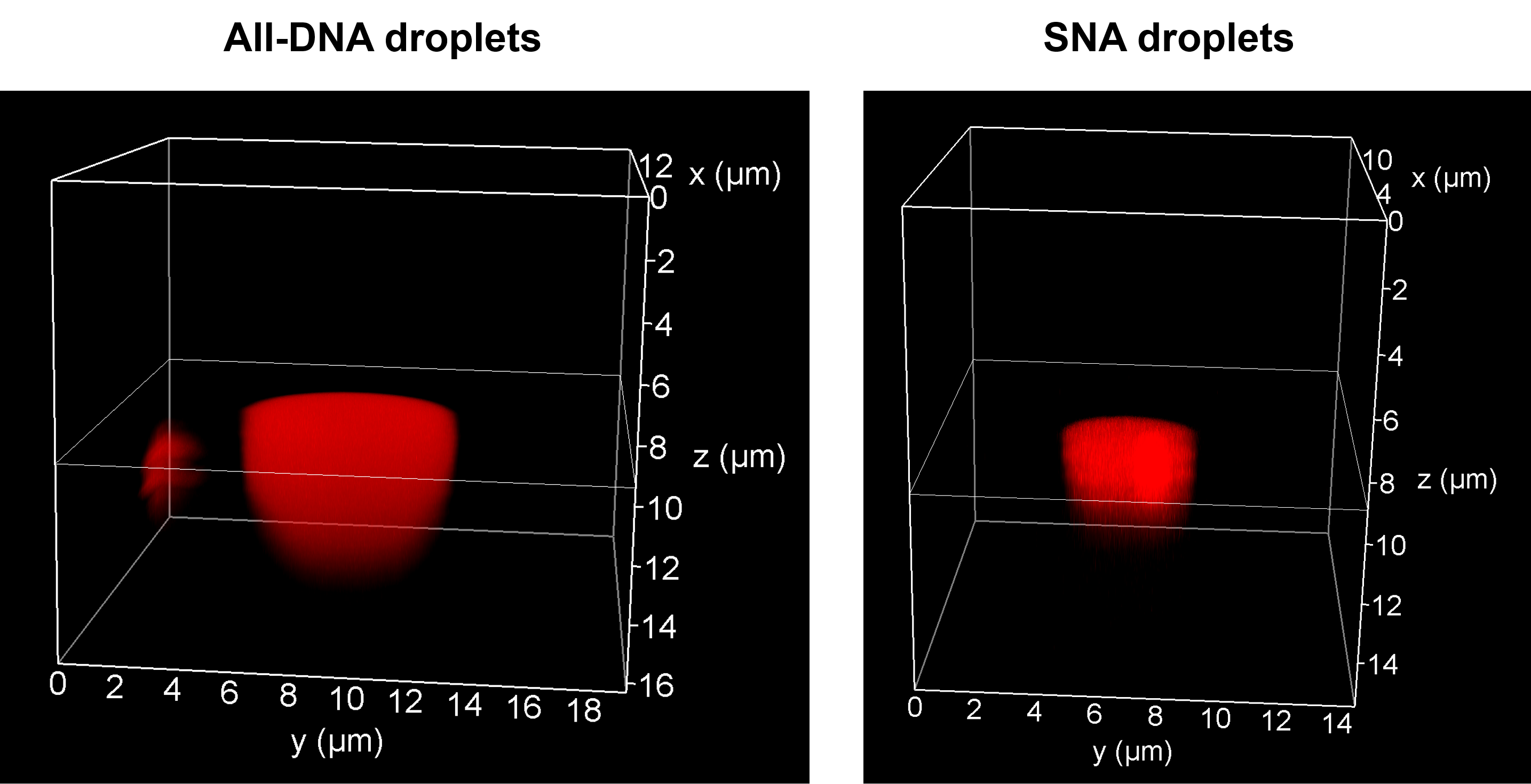
**

**Figure S21.** Three-dimensional cross-sectional CLSM images of all-DNA and SNA droplets.

**
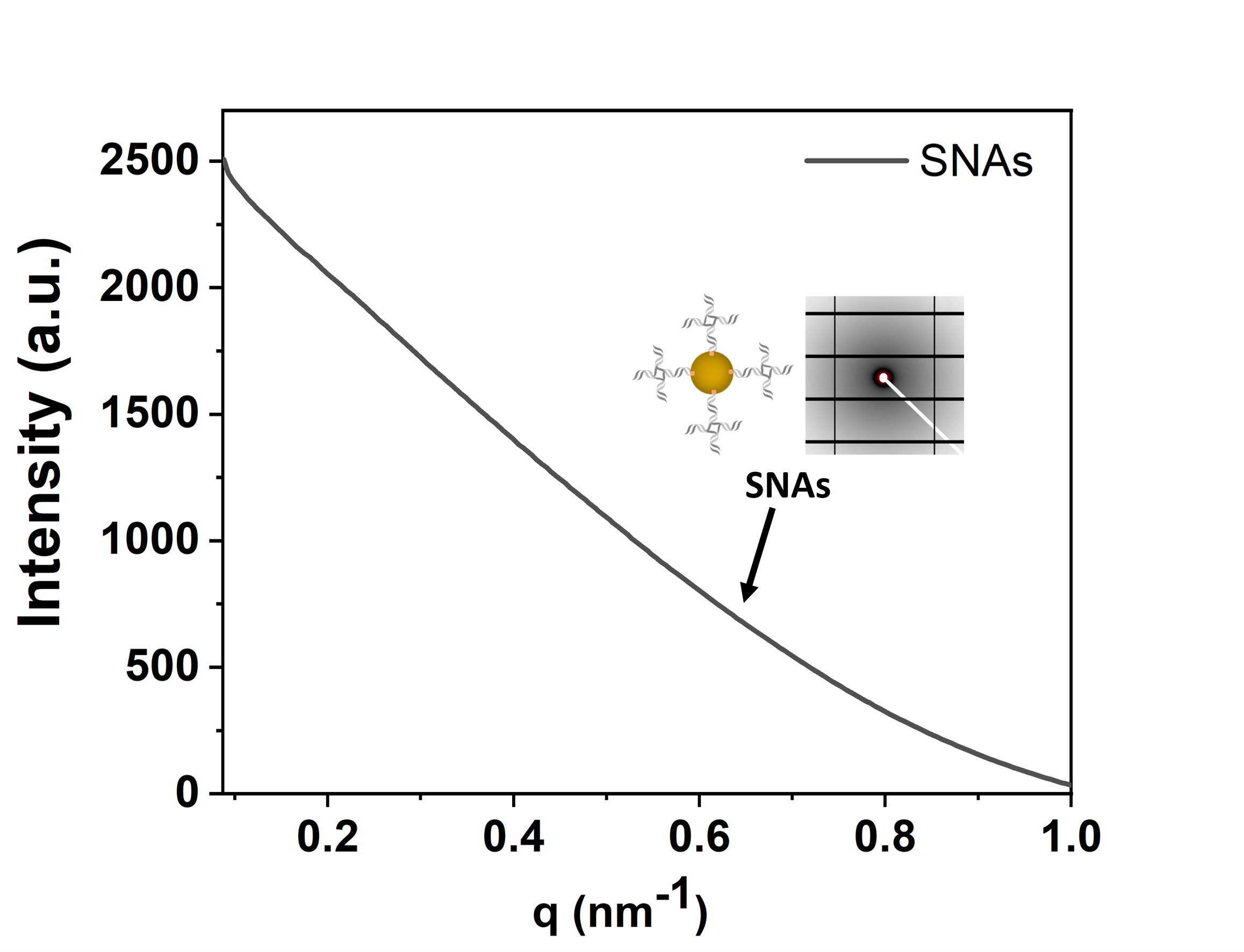
**

**Figure S22.** The scattering curves of SNAs, with insets showing the corresponding two-dimensional scattering patterns.

**
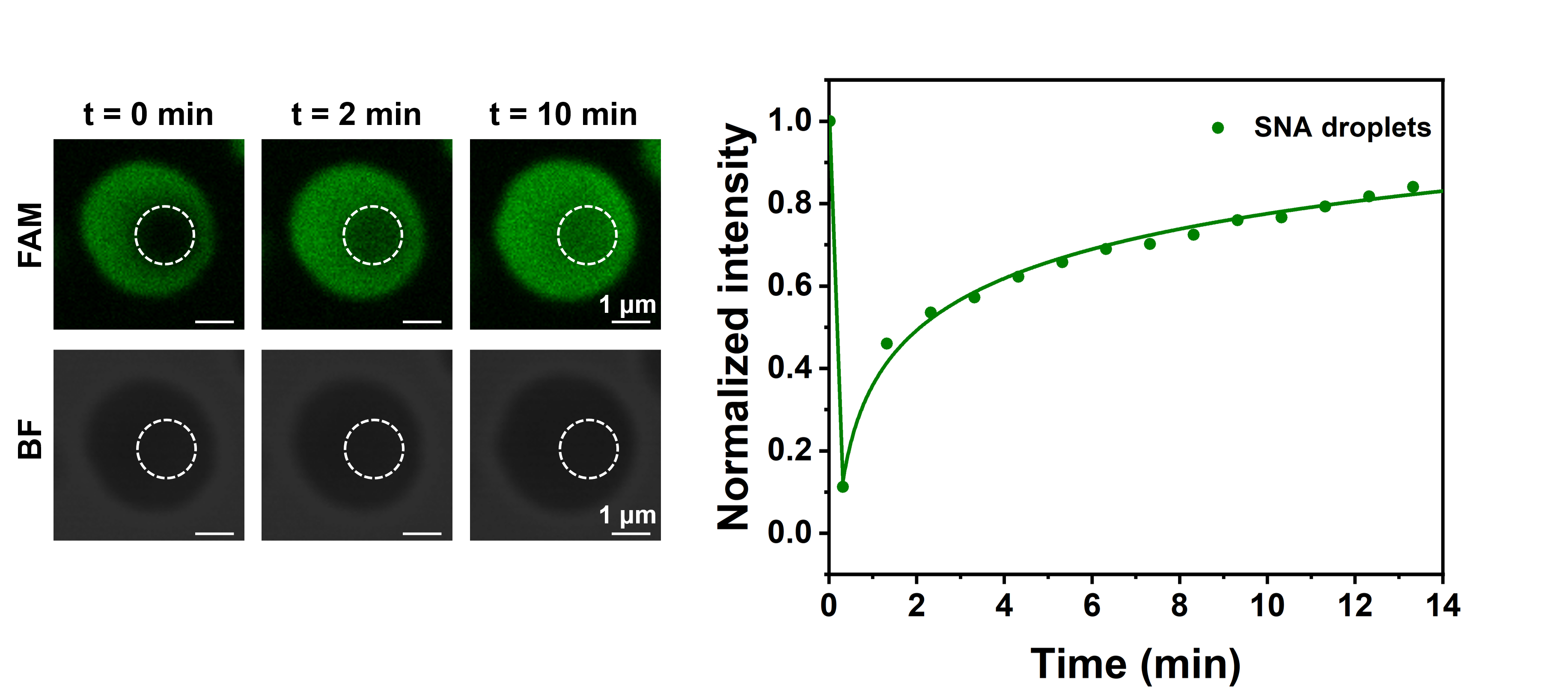
**

**Figure S23.** Representative CLSM images (left) showing the fluorescence recovery from photobleached FAM-labeled SNA droplets at specific timepoints, and the corresponding fluorescence recovery curve (right). Scale bars: 1 μm.

**
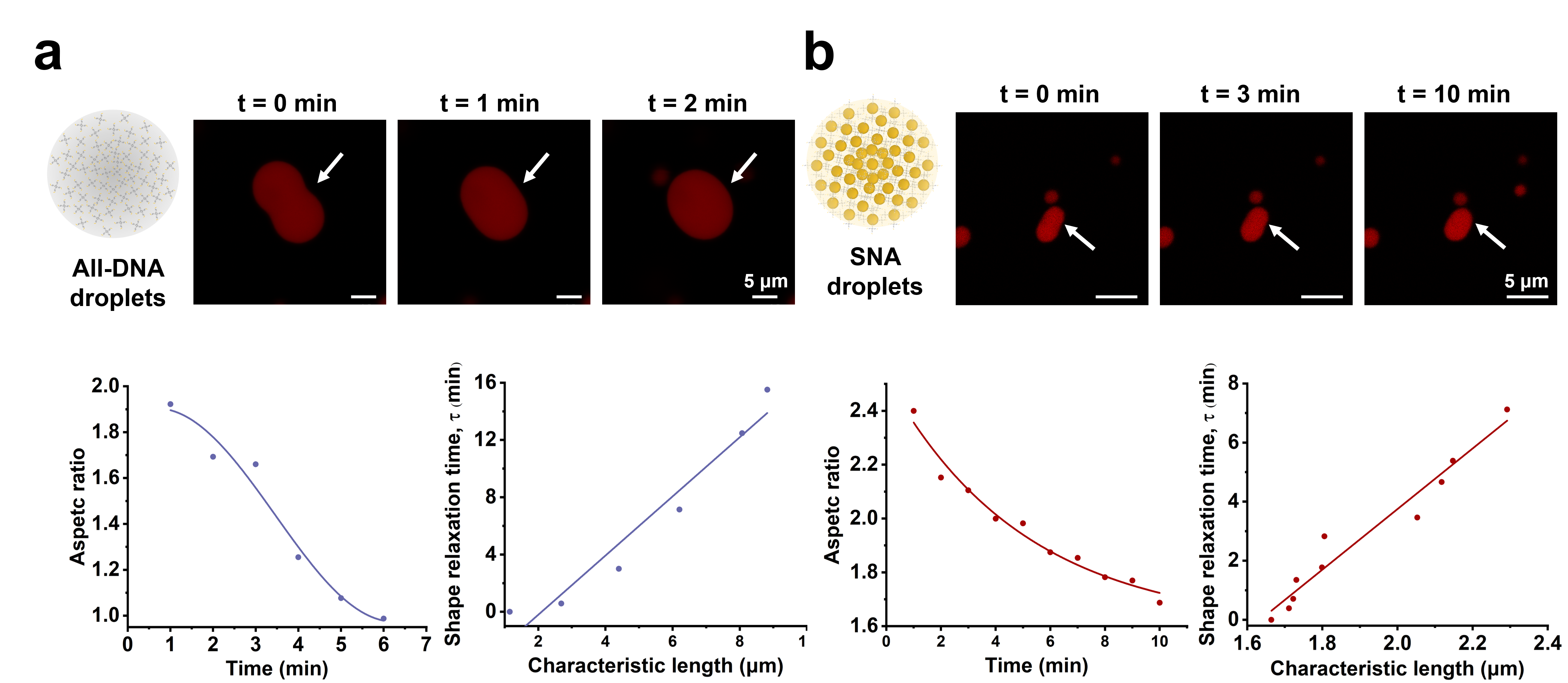
**

**Figure S24.** Top panel: CLSM images of the fusion process of all-DNA droplets (a) and SNA droplets (b) in 350 mM NaCl. Bottom panel: time-dependent changes in the aspect ratio (*AR*) of droplets during the fusion process. Data were fit with an exponential function to extract the shape relaxation timescale (*τ*). Relationship between shape relaxation time (*τ*) and droplet length scale (*l*). The slope of the linear fit corresponds to the inverse capillary velocity (*μ/γ*). All experiments were conducted at room temperature. Scale bar, 5 μm.

**
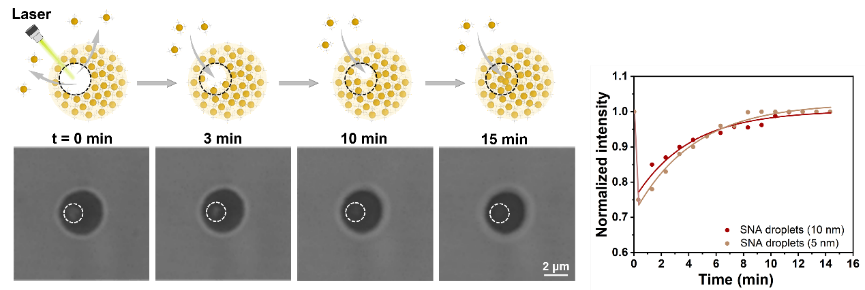
**

**Figure S25.** Representative CLSM images showing the photothermal characteristics and liquid-like moving behavior of SNA droplets. Statistical analysis results of the brightness of the circled area of SNA droplets, proving the detaching and recruiting process of SNAs after laser irradiation.

**
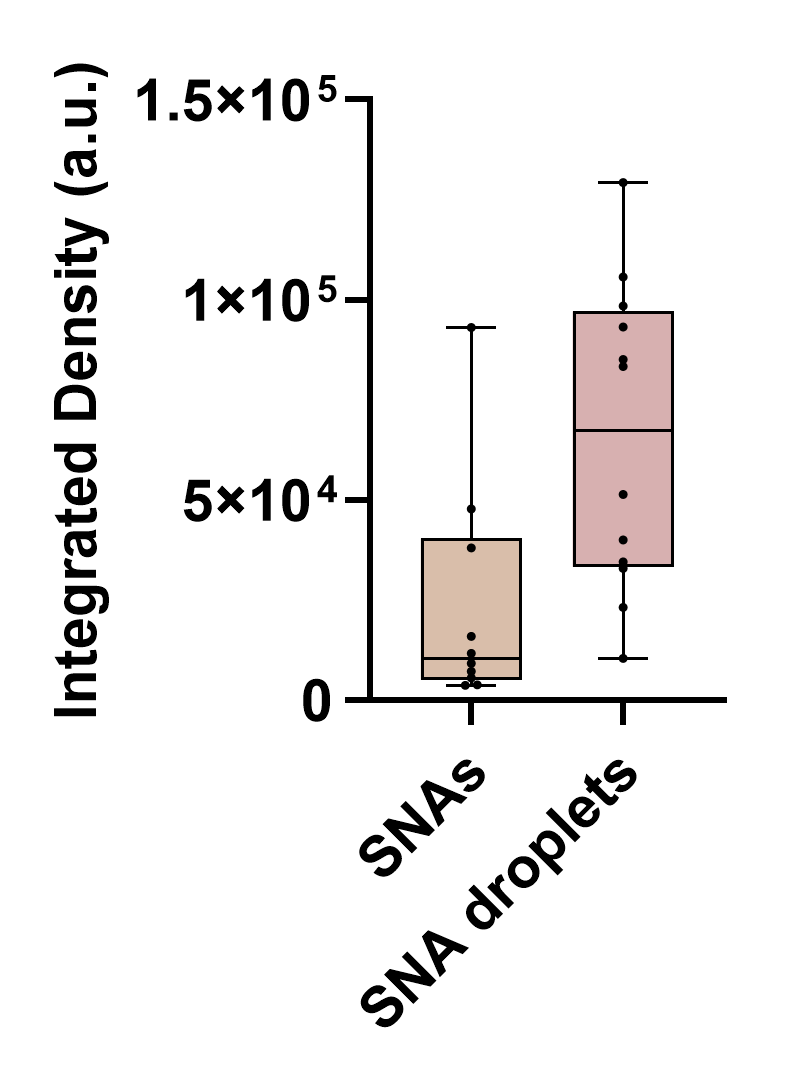
**

**Figure S26.** Analysis of the average fluorescence intensity to evaluate the cellular uptake efficiency of SNAs monomers or SNA droplets.


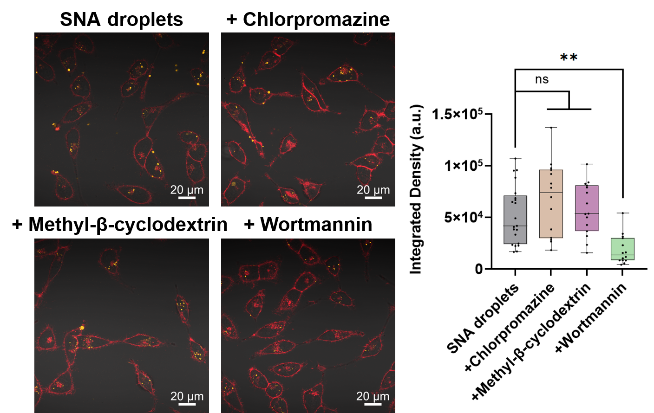


**Figure S27.** Representative confocal images of untreated HeLa cells and HeLa cells treated with different uptake inhibitors, along with statistical analysis of fluorescence intensity from more than 15 confocal images. The inhibitors used include Chlorpromazine (inhibitor of clathrin), Methyl-β-cyclodextrin (inhibitor of caveolin), and Wortmannin (inhibitor of macropinocytosis). Scale bars: 20 μm. **, P < 0.01, one-way ANOVA.


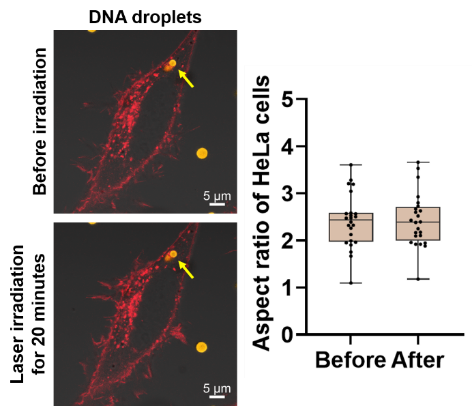


**Figure S28.** Representative CLSM images of HeLa cells incubated with all-DNA droplets before and after 20 minutes exposure to 561 nm laser. The laser-irradiated areas corresponding to the all-DNA droplets. Scale bars: 5 μm. Statistical analysis of the aspect ratio of twenty HeLa cells before and after treatments.


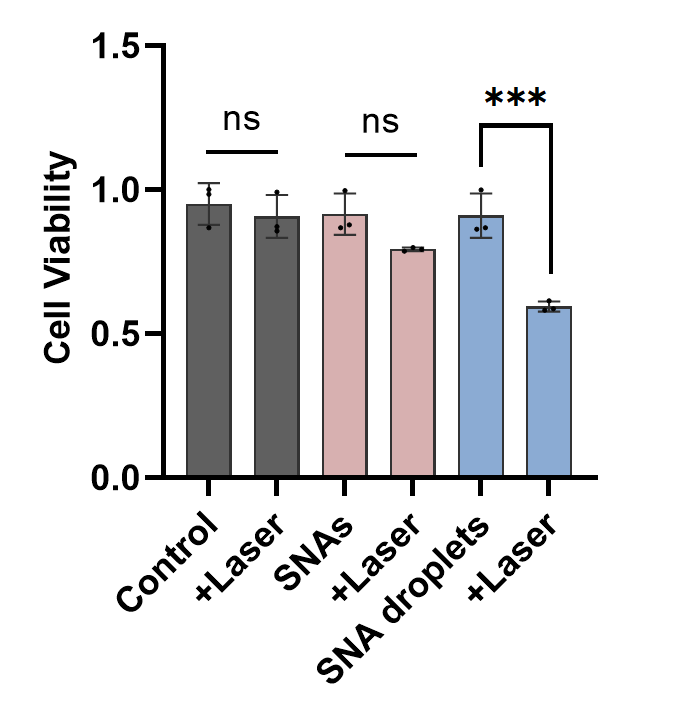


**Figure S29.** Cell viability was measured by CCK-8 in HeLa cells with blank control, 100 nM SNAs or SNA droplets before and after laser irradiation. Error bars represent standard deviation (SD) of three independent measurements, ***, P < 0.001, one-way ANOVA.

**Movie S1.** The three-dimensional structure reconstructed process of SNA droplets by STEM.

**Movie S2-S5.** Fluorescence recovery after photobleaching using CLSM for all-DNA droplets (Movie S2) and SNA droplets with 5 nm (Movie S3) and 10 nm (Movie S4) AuNPs, all assembled by SH-four-branched DNA. Movie S5 represents the recovery of SNA droplets (5 nm) in bright field. The photobleached droplets are highlighted with white boxes. Scale bars: 10 μm and 5 μm.

**Reference**

1. G. Yao *et al.*, Programming nanoparticle valence bonds with single-stranded DNA encoders. *Nature Materials* **19**, 781-788 (2019).

2. J. Martin, A Programmable Dual-RNA–Guided DNA Endonuclease in Adaptive Bacterial Immunity. *Science* **337**, 816-821 (2012).

3. C. Yao, R. Zhang, J. Tang, D. Yang, Rolling circle amplification (RCA)-based DNA hydrogel. *Nature Protocols* **16**, 5460-5483 (2021).

4. S. Ray *et al.*, α-Synuclein aggregation nucleates through liquid–liquid phase separation. *Nature Chemistry* **12**, 705-716 (2020).

5. Y.-W. Lee *et al.*, Direct Cytosolic Delivery of Proteins through Coengineering of Proteins and Polymeric Delivery Vehicles. *Journal of the American Chemical Society* **142**, 4349-4355 (2020).

6. Z. Chen, X. Chen, B. Zhao, H. Zhang, H. Zhang, Efficient Poly-Adenine-Tailed DNA Functionalization of Gold Nanorods for Tailored Nanostructure Assembly. *The Journal of Physical Chemistry Letters* **15**, 4400-4407 (2024).

7. Y. Sato, M. Takinoue, Sequence-dependent fusion dynamics and physical properties of DNA droplets. *Nanoscale Advances* **5**, 1919-1925 (2023).

8. S. Do, C. Lee, T. Lee, D.-N. Kim, Y. Shin, Engineering DNA-based synthetic condensates with programmable material properties, compositions, and functionalities. *Science Advances* **8**, eabj1771 (2022).

9. B.-j. Jeon *et al.*, Salt-dependent properties of a coacervate-like, self-assembled DNA liquid. *Soft Matter* **14**, 7009-7015 (2018).

10. G. Fabrini *et al.*, Co-transcriptional production of programmable RNA condensates and synthetic organelles. *Nature Nanotechnology* **19**, 1665-1673 (2024).

11. J. M. Stewart *et al.*, Modular RNA motifs for orthogonal phase separated compartments. *Nature Communications* **15**, 6244 (2024).

12. X. Chen *et al.*, PolyA-based DNA bonds with programmable bond length and bond energy. *NPG Asia Materials* **12**, 49 (2020).
